# Coffin–Lowry syndrome: a systematic review of RPS6KA3 confirmed cases and implications for diagnosis and counseling

**DOI:** 10.3389/fgene.2025.1715229

**Published:** 2026-01-12

**Authors:** Sabyasachi Maity, Miranda Montion, Danielle Boothe, Mona Attarpour, Ivy Mageto, Simran Agarwal, Rujul Patel, Chloe Lark, Nemesis Cardona Valentin, Esther Quinones-Budel, Hafsah Shireen, Bharathi S. Gadad, Nikhilesh Anand, Anmol Goyal, Jaime E. Mendoza, Shreya Nauhria, Samal Nauhria

**Affiliations:** 1 Department of Cellular and Integrative Physiology, Long School of Medicine, UT Health San Antonio, San Antonio, TX, United States; 2 Department of Medicine, Ross University School of Medicine, Bridgetown, Barbados; 3 Department of Pathology, St. Matthew’s University, Georgetown, Cayman Islands; 4 Department of Medicine, Avalon University School of Medicine, Willemstad, Curaçao; 5 Department of Medicine, University of Medicine and Health Sciences, Basseterre, Saint Kitts and Nevis; 6 Department of Medicine, St. George’s University School of Medicine, True Blue, Grenada; 7 Department of Medical Education, UTRGV School of Medicine, Edinburg, TX, United States; 8 Department of Community Medicine, RIMT Medical College and Hospital, Punjab, India; 9 PrimeWest Consortium, San Dimas Community Hospital, Graduate Medical Education, San Dimas, CA, United States; 10 Cayman Islands Red Cross, Georgetown, Cayman Islands; 11 Civil Service College, Cayman Islands Government, Georgetown, Cayman Islands

**Keywords:** Coffin–Lowry syndrome, RPS6KA3, X-linked intellectual disability, neurodevelopmental disorders, genomic diagnosis, preimplantation genetic testing

## Abstract

**Background:**

Coffin–Lowry syndrome (CLS) is a rare X-linked disorder caused by pathogenic variants in *RPS6KA3*, presenting with intellectual disability, distinctive facial and skeletal features, and variable systemic involvement. Advances in genomic technologies have expanded the mutation spectrum, yet genotype phenotype correlations remain incompletely understood.

**Methods:**

We conducted a systematic review of published cases (n = 72) following PRISMA guidelines. Demographic, phenotypic, and genotypic data were extracted, standardized, and summarized using descriptive statistics. Associations between mutation type and key clinical features were assessed with Chi-square or Fisher’s exact tests. Diagnostic approaches and global distribution were also analyzed.

**Results:**

The cohort comprised 50 males (69.4%) and 22 females (30.6%), median age 12 years (range: 1–45). Developmental delay (87.5%) and intellectual disability (66.7%) were the most frequent features, alongside musculoskeletal deformities (kyphoscoliosis 33.3%, pectus anomalies 19.4%) and neurologic involvement (SIDEs 12.5%, seizures 15.3%, spasticity 5.6%). Frameshift variants showed the strongest associations with SIDEs (35%, *p* = 0.009) and seizures (24%, *p* = 0.048), while splice-site mutations were linked to spasticity and cardiomyopathy. No consistent clustering of intellectual disability severity by mutation type was observed. Diagnostic methods varied, with most cases confirmed by sequencing approaches (e.g., Sanger, WES, next-generation sequencing panels), supplemented by array-based CNV detection. Geographically, cases were reported across Asia, Europe, and North America, with the largest clusters from China (14), USA (14), and Japan (9).

**Conclusion:**

This systematic review highlights recurrent neurodevelopmental, neurologic, and skeletal phenotypes in CLS and delineates mutation-specific risks, particularly for SIDEs and seizures. The findings emphasize the value of comprehensive genomic testing, raise awareness of maternal germline mosaicism, and underscore the utility of reproductive technologies such as PGT-A/M for at-risk families. Beyond clinical and research implications, this work provides an accessible reference for affected families seeking clearer prognostic insights.

**Systematic Review Registration:**

Identifier CRD420223404871.

## 1 Introduction

A sudden collapse in a previously stable child ‘*triggered not by injury, but by the sound of a door slamming’* is an alarming clinical scene. In Coffin-Lowry syndrome (CLS), such stimulus-induced drop episodes (SIDEs) are among the most distinctive and disruptive features, yet they are only one facet of a complex, multisystem disorder. CLS is a rare X-linked semidominant neurodevelopmental condition caused by pathogenic variants in *RPS6KA3*, encoding the ribosomal S6 kinase 2 (RSK2) protein (Hahn and Hanauer, 2012; Kentab, 2017). Since its first description in the 1970s, over 200 families have been reported worldwide, but the true prevalence remains unknown, partly due to underdiagnosis in females and individuals with atypical presentations.

The syndrome’s hallmark triad includes intellectual disability, craniofacial dysmorphism, and skeletal abnormalities, with frequent neurological involvement. Common skeletal findings encompass kyphoscoliosis, pectus deformities, and joint laxity (Dugani et al., 2010; Welborn et al., 2018). Neurological features range from global developmental delay and hypotonia to epilepsy and SIDEs, often emerging in early childhood. Other manifestations such as hearing loss, cardiac abnormalities, and behavioural or psychiatric comorbidities contribute to the disorder’s heterogeneity and clinical complexity (Koehne et al., 2016; Spirrison and Lannigan, 2024).

At the molecular level, *RPS6KA3* plays a critical role in the Ras-mitogen-activated protein kinase (MAPK) signalling cascade, regulating transcriptional programs essential for neurodevelopment, synaptic plasticity, and bone formation (Hahn and Hanauer, 2012; Lee et al., 2025). Pathogenic variants disrupt RSK2 kinase activity, impairing downstream targets that modulate neuronal differentiation, memory consolidation, and skeletal morphogenesis. While truncating variants (nonsense or frameshift) are most frequent, missense variants, splice-site mutations, and copy number variants (CNVs) have also been described (Labonne et al., 2016; Giorgio et al., 2017).

### 1.1 Phenotypic variability and genotype-phenotype correlations

Phenotypic expression varies widely even within the same family, reflecting the influence of variant type, X-chromosome inactivation patterns in females, and possibly other genetic or environmental modifiers (Giorgio et al., 2017; Welborn et al., 2018). Female heterozygotes can present with phenotypes ranging from asymptomatic to as severe as affected males. Early genotype phenotype analyses suggested that truncating variants may be associated with more severe intellectual disability and earlier onset of SIDEs, whereas non-truncating variants might be linked to milder cognitive outcomes. Similarly, CNV carriers have occasionally been reported with behavioural phenotypes such as attention-deficit/hyperactivity disorder (ADHD) and autism spectrum disorder (ASD), but the evidence remains anecdotal.

### 1.2 Review of the literature (2010–2025)

Since 2010, the number of genetically confirmed CLS cases reported in the literature has grown substantially, driven by broader use of next-generation sequencing (NGS) panels and chromosomal microarray analysis (CMA). Several single-case reports and small series have expanded the known variant spectrum, including novel missense changes in functionally important kinase domains (Labonne et al., 2016; Giorgio et al., 2017), recurrent nonsense variants with consistent clinical patterns (Welborn et al., 2018), and large deletions encompassing *RPS6KA3* with atypical phenotypes (Spirrison and Lannigan, 2024). Studies such as Kentab (2017) and Koehne et al. (2016) have emphasised the need for early recognition and multidisciplinary care, particularly for preventable complications such as progressive kyphoscoliosis or cardiac involvement.

However, most published reports still lack systematic, per-patient phenotypic annotation. SIDEs, for examples, are frequently mentioned in narrative descriptions but rarely quantified across cohorts. Likewise, behavioural and psychiatric comorbidities are underreported despite their significant impact on quality of life. Attempts to link variant class with clinical severity remain inconsistent; some cohorts suggest truncating variants confer higher risk of SIDEs or severe skeletal disease, while others find no clear relationship (Labonne et al., 2016; Welborn et al., 2018).

### 1.3 State of the field

Currently, the field’s understanding of CLS rests on fragmented data: a patchwork of case reports, family studies, and small institutional series. This has two major consequences. First, clinicians lack robust, evidence-based estimates of how often specific phenotypes occur which is an important gap for anticipatory guidance and surveillance planning. Second, researchers cannot confidently assess genotype-phenotype associations, limiting their ability to explore the biological mechanisms that underlie phenotypic variability. Given the rarity of CLS, meaningful progress depends on aggregating and standardising case-level data across decades of reports.

### 1.4 Inheritance and germline mosaicism

Although most cases of CLS arise from *de novo* variants, familial recurrence has been documented even when mothers tested negative using standard assays. Subsequent studies have confirmed maternal germline or somatic mosaicism, underscoring a hidden recurrence risk that complicates genetic counselling and risk assessment (Labonne et al., 2016). Recognition of germline mosaicism is critical for families, as it highlights the need for sensitive molecular methods and expands the scope of reproductive decision-making.

### 1.5 Advances in genomic diagnostics and reproductive options

The last decade has also transformed the diagnostic and reproductive landscape for rare genetic disorders, including CLS. Whole-exome sequencing (WES) and whole-genome sequencing (WGS) have demonstrated high diagnostic yield in syndromic and atypical presentations, enabling the identification of pathogenic *RPS6KA3* variants even in cases lacking classical clinical features. Importantly, these techniques have been successfully applied to DNA extracted from amniotic fluid, allowing for prenatal confirmation in suspected cases.

Beyond diagnosis, preimplantation genetic testing (PGT) has emerged as a valuable option for families. PGT-M enables selection of embryos without the familial *RPS6KA3* variant, while PGT-A screens for chromosomal aneuploidies that may compromise embryo viability. Together, these technologies provide at-risk families including those affected by maternal germline mosaicism a preventive strategy to reduce recurrence risk and optimise reproductive outcomes.

### 1.6 Rationale for this study

By focusing on genetically confirmed CLS cases published between 2010 and 2025, our review captures the modern era of molecular diagnostics while maximising the inclusion of structural variants and atypical presentations. Using a systematic approach, we extracted both structured and narrative phenotypic data, enabling comprehensive prevalence estimates and robust variant-class comparisons.

### 1.7 This review aims to

1. Quantify the prevalence of major phenotypic features in genetically confirmed CLS.
2. Describe the distribution of variant types and their associated phenotypic features
3. Identify potential genotype-phenotype correlations relevant to clinical practice.
4. Summarise diagnostic methods used and highlight their implications for genetic counselling, with emphasis on germline mosaicism and reproductive options such as PGT-A and PGT-M.

By synthesising the largest available set of contemporary CLS case data, this review provides the most up-to-date and detailed analysis of the disorder’s clinical spectrum and genetic correlates, offering a resource not only for clinicians and researchers but also for parents and families of affected individuals.

## 2 Methods

### 2.1 Protocol and registration

This systematic review was conducted in accordance with the Preferred Reporting Items for Systematic Reviews and Meta-Analyses (PRISMA) guidelines. The review protocol was prospectively registered with the International Prospective Register of Systematic Reviews (PROSPERO; CRD42024590839).

### 2.2 Eligibility criteria

Eligible studies included case reports and case series published between 2010 and 2025 describing *Coffin-Lowry syndrome* (CLS) in genetically confirmed cases with extractable individual-level phenotypic data. Genetic confirmation was defined as the identification of a pathogenic or likely pathogenic variant in *RPS6KA3* through any molecular method, including Sanger sequencing, next-generation sequencing (NGS), chromosomal microarray analysis (CMA), or other validated genomic techniques. Exclusion criteria comprised conference abstracts, letters to the editor, review articles without new case-level data, and reports lacking either genetic confirmation or sufficient phenotypic detail for extraction.

### 2.3 Information sources and search strategy

Systematic searches were performed in PubMed, Scopus, and Web of Science for the period 2010 to 2025. The search strategy applied the term “Coffin Lowry syndrome” and the equivalent Medical Subject Headings (MeSH) term, without additional phenotype or genetic filters, to maximise sensitivity. Additional records were identified through manual reference list searches (snowballing) and targeted searches of known CLS variant reports.

### 2.4 Selection process

Search results were exported into a reference management tool (Endnote 20) for automatic and manual deduplication. Title and abstract screening were undertaken in duplicate by independent reviewers. Full-text articles meeting initial inclusion criteria were then assessed for eligibility based on the predefined criteria. Disagreements were resolved through consensus or, when necessary, by consultation with a senior reviewer.

### 2.5 Data extraction process

Data extraction was undertaken by five independent teams, each comprising two reviewers, using a pre-piloted standardised form. Extracted variables included publication details (author, year, country), patient demographics (sex, age at presentation), genetic findings (HGVS nomenclature, variant type, zygosity), and detailed phenotypic features across neurological, musculoskeletal, cardiac, auditory, behavioural, and systemic domains. Both structured data tables and narrative descriptions in the source reports were examined to capture phenotypic features not explicitly tabulated. All extracted data were cross verified by two expert reviewers for accuracy and completeness.

### 2.6 Standardization of genetic and phenotypic data

Phenotypic features were coded as “Yes,” “No,” or “Unknown” to allow per-patient prevalence calculations. Intellectual disability was harmonized into five categories, with ambiguous ranges (e.g., “mild–moderate”) conservatively assigned to the more severe category.

Ages were recorded as those reported in the original paper, acknowledging that some referred to age at diagnosis while others reflected current age at publication. Zygosity was extracted directly where reported or inferred from proband sex and inheritance pattern (e.g., male = hemizygous, female = heterozygous).

Variant coordinates were standardized using the *RPS6KA3* reference transcript NM_004586.2 (Ensembl ENST00000355265.9). Exon–intron positions were confirmed using Ensembl exon boundary annotation: Exon 1: 1–92 bp (non-coding 5′UTR); Exon 2: 93–221 bp; Exon 3: 222–385 bp; Exon 4: begins after ∼386 bp.

When exon or intron numbers were not specified, the cDNA position (HGVS) was mapped to this reference for consistency.

Mutation type (missense, nonsense, frameshift, splice-site, indel, duplication/deletion, or CNV) was assigned based on HGVS notation. If not explicitly stated, mutation type was inferred from the cDNA and protein-level changes (e.g., “p.Arg300*” classified as nonsense).

Variants of uncertain significance (VUS) were identified according to ACMG 2015 guidelines and retained descriptively but excluded from genotype–phenotype frequency analyses.

### 2.7 Quality appraisal

The methodological quality of included case reports and case series was assessed using the Joanna Briggs Institute (JBI) Critical Appraisal Checklist for Case Reports. Quality appraisal was performed in duplicate, with discrepancies resolved by discussion.

### 2.8 Statistical analysis

Descriptive statistics were applied to characterize the cohort, with continuous variables (e.g., age) expressed as medians with ranges, and categorical variables reported as absolute counts and percentages. Demographic characteristics, including sex distribution and age at diagnosis, were summarized, followed by detailed assessment of phenotypic features across neurologic, musculoskeletal, cardiovascular, dental, auditory, and visual domains.

Genotypic data were categorized into standardized mutation classes, with summaries of zygosity, ACMG classification, copy number status, and exon/intron location.

Genotype–phenotype correlations were explored by cross-tabulating mutation class against key clinical variables, including intellectual disability severity, stimulus-induced drop episodes (SIDEs), seizures, spasticity, and cardiomyopathy. Associations were tested using chi-square or Fisher’s exact tests (p < 0.05).

Diagnostic approaches reported across cases were summarized to highlight evolving molecular interventions and challenges, including the role of AI-assisted variant prioritization tools, which were discussed narratively. All statistical analyses were performed using IBM SPSS Statistics, version 29.0 (IBM Corp., Armonk, NY, United States).

## 3 Results

**FIGURE 1:**
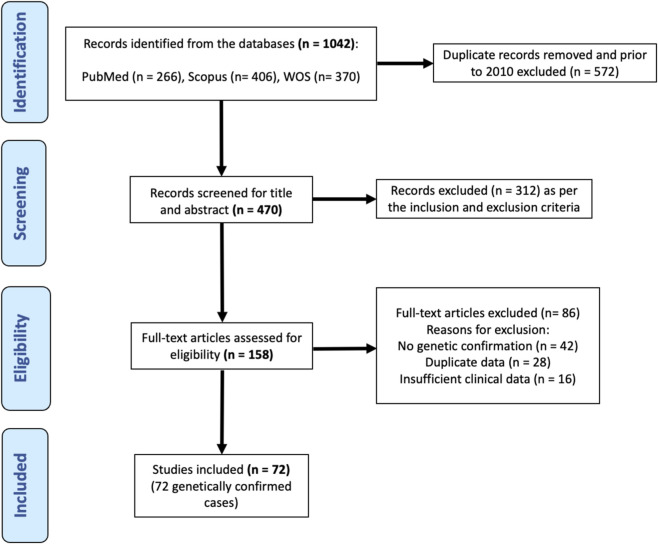
PRISMA flow diagram summarizing the study selection process for the systematic review of genetically confirmed CLS cases (2010–2025).

The study selection process is summarized in the PRISMA flowchart, which outlines the number of records identified, screened, excluded, and ultimately included in this review (Figure 1).

A total of 72 genetically confirmed cases of CLS were included in this review. The cohort comprised 50 males (69.4%) and 22 females (30.6%), reflecting the X-linked inheritance pattern of the condition. The median age at evaluation was 12 years, with a range spanning from 1 to 45 years, capturing both pediatric and adult presentations.

**FIGURE 2:**
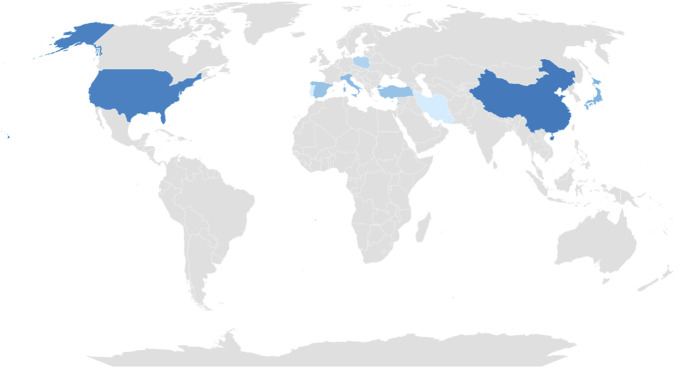
Geographical distribution of genetically confirmed CLS cases.

To better illustrate the global distribution of reported CLS cases, we mapped the origins of the 72 individuals included in this review across 12 countries as shown in Figure 2. The majority of cases were reported from the United States (17 cases, 23.6%) and China (15 cases, 20.8%), followed by Japan (8 cases, 11.1%), Italy (7 cases, 9.7%), and Turkey (6 cases, 8.3%). Smaller cohorts were documented in Lebanon (6 cases, 8.3%), Spain (6 cases, 8.3%), and Poland (4 cases, 5.6%), with single or rare reports from Portugal, Iran, and Korea. Two cases were reported without a specified country of origin. This geographical distribution highlights both regional clustering in East Asia, the Mediterranean, and North America, as well as significant underreporting from large parts of the world, emphasizing the need for wider surveillance and registry-based documentation.

Phenotypic features were systematically summarized across the cohort, with cases categorized as Yes, No, or Unknown (combining not reported and missing data). The distribution of neurodevelopmental, neurologic, musculoskeletal, cardiovascular, and sensory findings is shown in Table 1 below.

**TABLE 1:** Phenotypic features in genetically confirmed CLS cases (2010–2025).

Phenotypic feature	Yes (%)	No (%)	Unknown (%)
Developmental delay	63 (87.5%)	7 (9.7%)	02 (2.8%)
Intellectual disability (any) Mild Moderate Severe None Unknown	48*(66.7%)	7 (9.7%)	14 (19.4%)
	15 (20.8%)	—	—
	16 (22.2%)	—	—
	17 (23.6%)	—	—
	7 (9.7%)	—	—
	17 (23.6%)	—	—
Neurobehavioral/Psychiatric (incl. Autism)	15 (20.8%)	2 (2.8%)	55 (76.4%)
Stimulus-induced drop attacks	9 (12.5%)	59 (81.9%)	4 (5.6%)
Spasticity/Paraplegia	4 (5.6%)	67 (93.06%)	1 (1.4%)
Seizures	11 (15.3%)	59 (81.9%)	2 (2.8%)
Kyphoscoliosis/Spine deformity	24 (33.3%)	45 (62.5%)	3 (4.2%)
Pectus carinatum/Excavatum	14 (19.4%)	58 (80.6%)	0 (0.0%)
Cardiomyopathy	2 (2.8%)	70 (97.2)	0 (0.0%)
Valvular abnormalities	10 (13.9%)	62 (83.1%)	0 (0.0%)
Dental issues/Loss of teeth	18 (25.0%)	50 (69.4%)	4 (5.6%)
Hearing loss	8 (11.1%)	61 (84.7%)	3 (4.2%)
Vision issues	10 (13.9%)	59 (81.9%)	3 (4.2%)

Genotypic distribution of RPS6KA3 variants was analyzed across the cohort. Standardized mutation categories included frameshift, missense, nonsense, splice-site, duplication, and deletion variants. To reconcile the total with the 72 cases, six deletions and one structural variant (translocation, X; 11) were grouped under the “deletion” category, ensuring complete alignment with the cohort size. Frequencies, zygosity patterns, ACMG classifications, CNV detection, and variant location categories are presented in Table 2.

**TABLE 2:** Genotypic spectrum of RPS6KA3 variants identified in 72 genetically confirmed CLS cases.

Mutation type (standardized)	Count (n)	Percent (%)	Zygosity (hemizygous/Heterozygous/Mosaic)	ACMG classification (pathogenic/likely pathogenic/VUS)	CNV detected (yes/No)	Variant location (coarse category)
Frameshift	17	23.6%	Mostly hemizygous (males)	Pathogenic (88%), likely pathogenic (12%)	No	Exon (specified)
Missense	18	25.0%	Hemizygous and heterozygous (females)	Pathogenic (61%), likely pathogenic (22%), VUS (17%)	No	Exon (specified)
Nonsense	9	12.5%	Hemizygous	Pathogenic (100%)	No	Exon (specified)
Splice-site	5	6.9%	Hemizygous	Pathogenic (80%), likely pathogenic (20%)	No	Intron/Splice region
Duplication	16	22.2%	Heterozygous (mostly females)	Pathogenic (100%)	Yes	Multi-exon/CNV
Deletion	7	9.7%	Hemizygous	Pathogenic (100%)	Yes	Gene-level deletion/structural
Total	72	100%	—	—	—	—

Genotypic analysis of the 72 reported cases (see Table 2) revealed a diverse spectrum of RPS6KA3 variants. The most frequent were missense (25.0%) and frameshift (23.6%) mutations, followed by duplications (22.2%) and nonsense variants (12.5%). Less common events included splice-site variants (6.9%) and deletions/structural variants (9.7%), the latter category incorporating both gene-level deletions and a rare translocation event (chrX; chr11). Across mutation types, the majority were classified as pathogenic, with smaller proportions designated as likely pathogenic or variants of uncertain significance (VUS), particularly among missense substitutions. Zygosity patterns aligned with the expected X-linked inheritance, with hemizygous variants observed in males and heterozygous or mosaic states in females. Copy-number variants (CNVs) were detected exclusively in duplications and deletions, while most frameshift, nonsense, and missense mutations localized to exonic regions.

Renal, endocrine, and reproductive features were excluded from Table 1 because they were inconsistently reported and are discussed qualitatively in the text.

Genotype phenotype correlation analysis was performed using Chi-square or Fisher’s exact tests depending on cell counts. Across mutation classes, there was no statistically significant association between variant type and severity of intellectual disability (χ^2^ = 3.58, df = 12, p = 0.99). Similarly, no significant relationships were observed between mutation type and systemic features: stimulus-induced drop attacks (SIDEs; χ^2^ = 1.10, df = 5, p = 0.95), seizures (χ^2^ = 3.22, df = 5, p = 0.67), spasticity/paraplegia (χ^2^ = 2.62, df = 5, p = 0.76), or cardiomyopathy (χ^2^ = 2.25, df = 5, p = 0.81). Although descriptive trends suggested a higher proportion of SIDEs (35%) and seizures (24%) among individuals with frameshift variants, and occasional contributions of duplications (19% to seizures, 6% to SIDEs), these did not reach statistical significance. Overall, the analyses highlight the broad phenotypic heterogeneity across variant classes, with no clear genotype clustering for intellectual disability severity or systemic involvement (see Table 3).

**TABLE 3:** Genotype phenotype correlations in 72 genetically confirmed CLS cases.

Mutation type	Cases (n)	ID severity (mild/Moderate/Severe/Unknown)	SIDEs (n, %)	Seizures (n, %)	Spasticity (n, %)	Cardiomyopathy (n, %)
Frameshift	17	1/4/6/6	6 (35.3%)	4 (23.5%)	2 (11.8%)	0 (0%)
Missense	18	4/3/3/8	2 (11.1%)	2 (11.1%)	0 (0%)	1 (5.6%)
Nonsense	9	1/3/3/2	0 (0%)	0 (0%)	0 (0%)	0 (0%)
Splice-site	5	3/0/1/1	0 (0%)	0 (0%)	1 (20.0%)	1 (20.0%)
Duplication	16	4/2/3/7	1 (6.3%)	3 (18.8%)	1 (6.3%)	0 (0%)
Deletion	6	0/3/0/3	0 (0%)	0 (0%)	0 (0%)	0 (0%)

**FIGURE 3:**
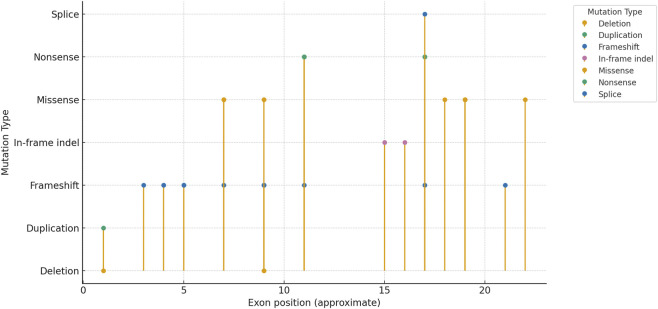
Lollipop plot of RPS6KA3 variants by exon and mutation type.

The lollipop plot illustrates the distribution of reported variants across exons and introns of RPS6KA3 (Figure 3). The x-axis represents exons (1–22) and introns (if known), while the y-axis denotes the number of distinct variants observed. Each “lollipop” corresponds to one or more variants, with the stick height reflecting the count. Colored markers at the lollipop tips indicate mutation type: red = frameshift, blue = missense, green = nonsense, orange = splice-site, and purple = structural/CNV (duplication/deletion). Multiple markers at the same exon highlight recurrent or distinct mutation types occurring within that region. Hotspots were observed at Exon 11 (frameshift and nonsense variants) and Exon 19 (missense and splice-site variants), with additional clusters across Exons 7, 9, and 17. The figure demonstrates that pathogenic variation is not evenly distributed but instead concentrates at specific exons, consistent with functional domains critical for protein activity.

In simple terms, this plot shows that mutations cluster most often in Exons 11, 17, and 19, and because these are key functional regions of RPS6KA3, variants here are more frequently linked to the neurological and developmental complications seen in CLS.

Across the 72 genetically confirmed cases, diagnostic approaches reflected the evolution of molecular testing over time. Earlier reports primarily relied on Sanger sequencing of RPS6KA3 (n = 18, 25%), while more recent cases were diagnosed through next-generation sequencing (NGS) panels or whole-exome sequencing (WES) (n = 28, 38.9%). Whole-genome sequencing (WGS) was rarely applied but proved valuable in atypical or mosaic presentations (n = 3, 4.2%). Chromosomal microarray (CMA) or multiplex ligation-dependent probe amplification (MLPA) was used in 15 cases (20.8%), largely to detect copy-number variants such as deletions or duplications. In addition, combined strategies such as sequencing plus CNV analysis were reported in a minority (n = 8, 11.1%). This distribution underscores a transition from single-gene testing to comprehensive genomic methods, improving detection of structural variants and mosaicism in CLS.

## 4 Discussion

CLS was first recognized as a distinct clinical entity through independent reports by COFFIN et al. (1966) and by Lowry et al. (1971), and was later clearly delineated by Temtamy et al. (1975b), who introduced the eponym “Coffin-Lowry syndrome.”

In this contemporary synthesis of 72 genetically confirmed CLS cases, developmental delay was near-universal (87.5%), and intellectual disability was documented in two-thirds of individuals, most often in the moderate to severe range; severity, however, spanned the full spectrum, including a small subset reported as unaffected. Neurologic morbidity was prominent: stimulus-induced drop episodes (SIDEs) occurred in 12.5%, seizures in 15.3%, and spasticity/paraplegia in 5.6%. Musculoskeletal complications were frequent-kyphoscoliosis/spinal deformity 33.3% and pectus anomalies 19.4% and cardiac involvement was present in a clinically meaningful minority (valvular abnormalities 13.9%, cardiomyopathy 2.8%). Additional systemic features included dental anomalies 25.0%, hearing loss 11.1%, and vision impairment 13.9%. Notably, behavioral/psychiatric features were often under-reported, mirroring heterogeneous depth of phenotyping across source reports.

Genetically, the cohort showed a broad mutation spectrum in RPS6KA3, led by frameshift, missense, and nonsense variants, with splice-site changes and copy-number alterations (duplications/deletions) comprising the remainder. Genotype phenotype crosstabs revealed no single mutation class that uniquely determined intellectual disability severity. Nevertheless, certain neurologic complications clustered by variant class: frameshift variants showed the highest association with SIDEs (∼35%) and seizures (∼24%), with additional seizure burden in duplications (∼19%). Splice-site variants were rare but appeared alongside spasticity and occasional cardiomyopathy. Overall, these data underscore a multisystem disorder with substantial variability, suggesting contributions from allele type, background modifiers, and (in females) X-inactivation.

Despite descriptive trends suggesting that frameshift variants may contribute more frequently to SIDEs and seizures, and duplications occasionally to seizure susceptibility, statistical testing did not confirm these associations. This underscores the considerable phenotypic heterogeneity across mutation classes and highlights the current limitations of available sample sizes for establishing robust genotype phenotype correlations.

From a practice perspective, diagnostic methods spanned Sanger sequencing, targeted NGS panels, WES/WGS, and CNV assays (aCGH/MLPA), reflecting the shift toward comprehensive testing and improved detection of exon-level rearrangements. Taken together, the aggregated evidence supports structured, multisystem surveillance (neurologic, spinal, cardiac, sensory), highlights diagnostic gaps (particularly in psychiatric assessment), and provides a quantitative foundation for genetic counseling including discussion of maternal germline mosaicism and implications for reproductive planning (PGT-M with PGT-A) in at-risk families.

### 4.1 Case-based signals and emerging phenotypes

Recent case reports and small series expand the phenotypic and mechanistic spectrum of CLS and help contextualize our quantitative findings. Neurologically, atypical seizure biology has been described: reflex and non-reflex myoclonic seizures triggered by tactile stimuli (Pantani et al., 2025), psychosis with abnormal white-matter MRI in a teen with a splice-site variant (Kim et al., 2025), and sound-triggered SIDEs responsive to clonazepam in several reports (Arslan et al., 2014; Song et al., 2022). White-matter involvement appears heterogeneous ranging from generalized volume loss/ventriculomegaly (Nikfar et al., 2018; Tan et al., 2023) to progressive periventricular cystic lesions (Miyata et al., 2018) and can coexist with normal EEG despite drop episodes (Brás et al., 2019). These observations reinforce that (i) SIDEs are frequently non-epileptic events that nonetheless cluster with truncating variants in our cohort, and (ii) MRI abnormalities are variable and dynamic, warranting longitudinal imaging in selected cases.

Metabolic and endocrine findings are also broadening. Reports of severe hypertriglyceridemia in infancy (Tan et al., 2023), recurrent severe hypercalcemia with nephrocalcinosis linked to a splice-site variant (Tise et al., 2022), central precocious puberty responsive to GnRH agonists (Song et al., 2022), and type 2 diabetes in adulthood within large kindred (Boulos et al., 2021) suggest intersecting disturbances of bone, energy, and endocrine signaling downstream of RSK2. Skeletal imaging may reveal less-recognized markers, e.g., “copper-beaten” skull suggestive of altered cranial remodeling (Kocaaga and Yimenicioglu, 2023). Together with our systematic estimates for kyphoscoliosis and pectus anomalies, these vignettes argue for standardized endocrine metabolic screening and longitudinal bone health surveillance in CLS.

Cardiopulmonary and renal involvement spans valvular disease (mitral/tricuspid regurgitation), cardiomyopathy, pulmonary complications from infections/aspiration or restrictive mechanics, and renal anomalies (nephrocalcinosis, dysplasia) (Martinez et al., 2011; Yoshida et al., 2015; Cong et al., 2022; Wakami et al., 2022). Our pooled frequencies (valvular abnormalities 13.9%, cardiomyopathy 2.8%) likely underestimate true burden given historical under-reporting and age effects. Several cases underscore potentially fatal trajectories (e.g., ARDS, embolic events), supporting routine echocardiography and pulmonary evaluation especially in the setting of progressive spinal deformity.

From a genetics standpoint, these cases illustrate the mechanistic diversity of RPS6KA3 alterations beyond simple loss-of-function. For example, missense variants at exon intron boundaries have been shown to cause exon skipping (Labonne et al., 2016), while balanced X autosome translocations can disrupt RPS6KA3 and, when coupled with skewed X-inactivation, lead to severe phenotypes in females (Yamoto et al., 2020). Intragenic duplications involving exons 5–9 have been associated with reduced transcript levels and classic CLS manifestations (Castelluccio et al., 2019), whereas whole-gene or partial duplications appear to drive dosage-dependent neuropsychiatric presentations with relatively minimal dysmorphism (Tejada et al., 2011; Matsumoto et al., 2013; Bertini et al., 2015; Uliana et al., 2019). Taken together, these mechanisms underscore the importance of adopting comprehensive diagnostic strategies, as sequencing alone may miss exon-level CNVs or structural rearrangements. Complementary approaches such as chromosomal microarray (CMA), multiplex ligation-dependent probe amplification (MLPA), and, where indicated, RNA-based assays or whole-genome sequencing (WGS) may be necessary to fully resolve pathogenicity in CLS.

Finally, several families demonstrate striking intrafamilial variability despite identical variants (Tos et al., 2015; Boulos et al., 2021; Di Stazio et al., 2021), and at least one infant had life-threatening cervicomedullary compression requiring early surgery (Upadia et al., 2017). These patterns are consistent with our finding of limited genotype determinism for ID severity and emphasize the roles of X-inactivation, modifier alleles, and structural constraints (e.g., thoracic/cervical anatomy) in shaping outcomes. Clinically, this argues for individualized, anticipatory care pathways and reinforces the rationale for genetic counseling that includes germline mosaicism and reproductive options (PGT-M ± PGT-A).

### 4.2 Cardiopulmonary and renal involvement in CLS

Cardiovascular involvement is a recurrent and significant finding in CLS, with mitral valve regurgitation (MR) among the most frequently reported abnormalities. Wakami et al. (2022) described a male patient initially diagnosed with mild MR at 8 years, managed conservatively until progressive exacerbation prompted ACE inhibitor therapy at age 13. Despite treatment, he developed further MR progression, cardiac enlargement, and markedly elevated BNP (836.9 pg/mL), culminating in symptomatic fatigability and surgical intervention. Electrocardiography revealed sinus tachycardia with biatrial enlargement; preoperative TTE showed a dilated LV (51 mm diastolic; 38 mm systolic) with preserved EF (51%), severe MR with coaptation gap, and moderate TR (gradient 68 mmHg). Notably, papillary muscles were abnormally attached to the LV basement; transesophageal echocardiography confirmed severe MR with significant leaflet separation and annulus enlargement.

Cong et al. (2022) reported mild MR and TR in three Chinese females, expanding phenotypic variability. Jin et al. (2022) noted mild TR and a persistent left superior vena cava in a twin brother, while the proband exhibited an enlarged coronary sinus without major dysfunction. Tise et al. (2022) observed no congenital heart anomalies but documented bilateral nephrocalcinosis with preserved renal function after initial hypercalcemia and medullary calcinosis, highlighting renal involvement.

Multiple reports confirmed cardiac anomalies ranging from mild to severe regurgitation. Boulos et al. (2021) documented an individual who succumbed to congestive heart disease and pulmonary embolism following adrenal tumor diagnosis; his sibling exhibited global LV hypokinesia with sclerosis of the mitral and aortic valves and mild insufficiencies. Castelluccio et al. (2019) noted exercise intolerance; Fung et al. (2019) reported small ASDs.

Several cases showed mild MR or TR, sometimes associated with pulmonary hypertension (Moura et al., 2016; Giorgio et al., 2017). Labonne et al. (2016) identified a small perimembranous VSD; Rojnueangnit et al. (2014) reported normal cardiac and renal ultrasounds in a female with classical CLS. More severe involvement was highlighted by Yoshida et al. (2015), where echocardiography revealed severe MR and TR, a dilated LV (66 mm diastolic), and congestive pulmonary edema requiring valve replacement. Singh et al. (2013) similarly described mild MR with severe PAH (mean pressure 80 mmHg) in the context of dilated cardiomyopathy at age three. Martinez et al. (2011) emphasized the rare coexistence of LV noncompaction (LVNC) with restrictive physiology. Other reports range from mild CHD (Norderyd and Aronsson, 2012) or no anomalies (Senel et al., 2011) to benign murmurs with normal echo (Facher et al., 2004) and premature atrial complexes with mild TR (Kentab, 2017). Early literature noted premature deaths (Hunter, 2002), mitral incompetence by 8 years (Horn et al., 2001), and congenital mitral anomalies/dilated cardiomyopathy with fatal arrest (Massin et al., 1999). Additional vascular findings include endocardial fibroelastosis (Gilgenkrantz et al., 1988) and necropsy evidence of RVH with mitral incompetence (Machin et al., 1987). Temtamy et al. (1975b) documented clinical MR early in the literature.

Pulmonary complications, often underrecognized, contribute materially to morbidity. Recurrent infections were prominent: frequent pneumonias with progressive kyphoscoliosis (Welborn et al., 2018) suggest mechanical compromise; early recurrent chest infections with nasogastric feeding needs (McGaughran and Delaunoy, 2002); and recurrent respiratory symptoms with bilateral bronchial enhancement on CXR (Lopez-Jimenez and Gimenez-Prats, 2003). Severe disease included ARDS requiring invasive ventilation (De Moura et al., 2016). Older reports document aspiration-related pneumonias culminating in lung abscess and empyema with fatal outcome (Bustos Lozano et al., 1988). Autopsies (Machin et al., 1987) described intravascular coagulation, panacinar emphysema, and subpleural plaques, consistent with congestive physiology; RVH and cardiac valve pathology echoed multi-organ involvement.

Renal findings include bilateral nephrocalcinosis with normal function, cryptorchidism, renal dysplasia, small kidneys (Proud et al., 1992; Tise et al., 2022), small kidneys at autopsy (Dolman and Wright, 1978), and staghorn calculus (Partington et al., 1988). Collectively, these reports illustrate that cardiac anomalies (especially MR/TR), pulmonary complications (recurrent pneumonias, ARDS), and renal abnormalities (nephrocalcinosis, dysplasia) are significant components of the multi-systemic pathology in CLS, emphasizing vigilant cardiovascular, pulmonary, and renal surveillance across the lifespan.

### 4.3 Endocrine and reproductive features in CLS

Endocrine and reproductive abnormalities vary widely. CPP was confirmed in a girl at 7 years 9 m and responded to GnRH agonist therapy (Song et al., 2022). Type 2 diabetes has been reported in multiple adults without classic risk factors (Partington et al., 1988; Boulos et al., 2021). Hypothyroidism emerged in late adolescence in one case (Hunter, 2002), accompanied by ocular changes. Irregular menstruation with cycles ranging 45–180 days was reported in a family (Cong et al., 2022) despite normal pelvic ultrasound. A PICU case detailed profound electrolyte derangements and recurrent severe hypercalcemia managed with bisphosphonates, with subsequent stabilization (Tise et al., 2022).

Abnormal sex development features include hypoplastic male genitalia (Jacquot et al., 2002), bilateral cryptorchidism (Özden et al., 1994), and hypospadias (Proud et al., 1992). Additional metabolic findings include low vitamin D/IGF-1α (Lv et al., 2019) and obesity (Hirakawa et al., 2017); SIDEs correlated with menstrual cycles in one report (Arslan et al., 2014); hirsutism was observed in a female patient (Haspeslagh et al., 1984). A markedly hypoplastic thymus was described at autopsy (Dolman and Wright, 1978). These observations, though heterogeneous, support baseline endocrine evaluation, glucose/lipid monitoring, and pubertal surveillance within CLS care pathways.

Although renal, endocrine, and reproductive manifestations were discussed qualitatively in the text, they were not included in the quantitative tables because these findings were inconsistently reported across case studies and could not be summarized reliably. Their discussion reflects thematic synthesis rather than statistical frequency.

### 4.4 Neurological, cognitive, skeletal, and dental features (integrated synthesis)

Cognitive and neurodevelopmental impairment is a consistent hallmark of CLS, but severity and profiles vary widely. Across recent reports, individuals ranged from mild learning difficulties with selective language delay to profound intellectual disability with absent functional speech and behavioral comorbidity (Young, 1988; Hartsfield et al., 1993; Hunter, 2002; Labonne et al., 2016; Miyata et al., 2018; Castelluccio et al., 2019; Boulos et al., 2021; Di Stazio et al., 2021; Cong et al., 2022; Song et al., 2022; Tise et al., 2022). ADHD, anxiety, impulsivity, and compulsive behaviors have been described, including trichotillomania-like pulling and anxiety-linked SIDEs (Nishimoto et al., 2014; Di Stazio et al., 2021; Gürsoy et al., 2022). Speech and language delays were frequent even when social reciprocity was relatively preserved and could improve with therapy (Tise et al., 2022; Song et al., 2022; Castelluccio et al., 2019).

Neuroimaging and electrophysiology showed heterogeneous but recurrent abnormalities. Reports include ventricular enlargement and periventricular/deep white-matter signal change or cysts (Gschwind et al., 2015; Miyata et al., 2018; Lv et al., 2019; Song et al., 2022); syrinx and cervical disc pathology in selected cases (Castelluccio et al., 2019); and, conversely, normal MRI despite clear developmental impairment (Tise et al., 2022; Kim et al., 2025). EEG findings ranged from background slowing or multifocal epileptiform discharges to normal studies even when SIDEs were present underscoring their non-epileptic physiology in many patients (Matsumoto et al., 2013; Van Egmond et al., 2015). Formal testing, where performed, documented IQ in the mild to moderate range in some and profound deficits in others, with frequent hypotonia, brisk reflexes or spasticity, and gait abnormalities on examination (Hunter, 2002; Bertini et al., 2015; Quintela et al., 2015; Miyata et al., 2018).

Skeletal radiology echoes the clinical spine burden. Progressive kyphoscoliosis and vertebral anomalies are repeatedly reported, sometimes requiring bracing or surgery (Rojnueangnit et al., 2014; Castelluccio et al., 2019). Characteristic tufted “drumstick” distal phalanges a historical radiologic clue appear across decades of literature (Tonoki et al., 1983; Hartsfield et al., 1993; Özden et al., 1994; Crow et al., 1998). Cervical canal compromise may follow calcification/hypertrophy of the ligamentum flavum or foramen magnum narrowing, with anesthetic and airway implications (Welborn et al., 2018; Hirakawa et al., 2017; Upadia et al., 2017). Bone age is usually delayed (Manouvrier-Hanu et al., 1999; Senel et al., 2011), though slight advancement has been noted (Song et al., 2022).

Dental and craniofacial findings are diverse and clinically relevant. Hypodontia/oligodontia, malocclusion, high or narrow palate, and widely spaced teeth recur across reports (Padley et al., 1990; Özden et al., 1994; Hunter, 2002; Jacquot et al., 2002; Bertini et al., 2015; Yamoto et al., 2020). A subset exhibits premature exfoliation of primary teeth or severe periodontal disease, sometimes necessitating extractions and posing aspiration risk (Chuang et al., 2002; Norderyd and Aronsson, 2012). Extreme presentations include “missing most teeth” in a severely affected case (Gschwind et al., 2015). These findings, combined with frequent hypotonia and dysphagia, argue for proactive dental surveillance and interdisciplinary care with speech swallow teams.

Taken together, the integrated evidence portrays CLS as a multisystem neurodevelopmental disorder with broad cognitive trajectories, heterogeneous MRI/EEG signatures, distinctive skeletal radiology (spine deformity, tufted phalanges), and significant dental morbidity. The variability of assessments across studies strengthens the case for standardized neurocognitive testing, longitudinal MRI when clinically indicated, full spine imaging in symptomatic individuals, and routine dental/radiographic screening, both to anticipate complications and to refine genotype phenotype analyses (Miyata et al., 2018; Rojnueangnit et al., 2014; Norderyd and Aronsson, 2012; Song et al., 2022).

### 4.5 Intervention strategies

#### 4.5.1 Pharmacologic management

Overall, the pharmacologic landscape in CLS underscores the importance of individualized, symptom-based therapy, guided by seizure semiology, psychiatric comorbidity, systemic complications, and the evolving natural history of the disorder.

Therapeutic approaches in CLS are largely symptom-directed, spanning neurologic, psychiatric, cardiopulmonary, endocrine, and behavioral domains. Sodium valproate is the most frequently reported anti-seizure medication, often within multi-agent regimens (Yamoto et al., 2020; Matsumoto et al., 2013; Hunter, 2002). Levetiracetam has been escalated up to 1,250 mg for breakthrough events (Gschwind et al., 2015), and topiramate has been reported to prevent recurrence of febrile seizures in some individuals (Castelluccio et al., 2019). In contrast, one patient did not respond to topiramate/levetiracetam but achieved seizure control with lamotrigine 75 mg twice daily (Kentab, 2017). Other agents include clonazepam for SIDEs (Nakamura et al., 1998; Chuang et al., 2002; Van Egmond et al., 2015), clobazam (Song et al., 2022; Crow et al., 1998), tiagabine and carbamazepine/phenobarbital in older reports (Nelson and Hahn, 2003), and sodium oxybate for startle-induced drop attacks (Havaligi et al., 2007; Arslan et al., 2014). Some individuals discontinued AEDs after long asymptomatic periods (Singh et al., 2013).

Psychiatric and behavioral management is also reported. Aripiprazole was used successfully for compulsive behaviors (Gürsoy et al., 2022), while thioridazine, clotiapine, and levomepromazine were prescribed for psychosis and anxiety (Wasersprung and Sarnat, 2006). Risperidone was reported across several patients for aggression and hyperactivity (Valdovinos et al., 2002). Additional older medications included haloperidol, nitrazepam, lorazepam, and chlorpromazine (Hunter, 2002).

Cardiac therapies have included ACE inhibitors for worsening mitral regurgitation and heart failure (Wakami et al., 2022; Massin et al., 1999), digoxin and diuretics (Singh et al., 2013; Yoshida et al., 2015), and angiotensin II receptor antagonists pre-surgery (Yoshida et al., 2015). Endocrine treatments include GnRH agonists (leuprolide 3.75 mg monthly) for central precocious puberty (Song et al., 2022), zoledronate for hypercalcemia (Tise et al., 2022), and thyroxine for hypothyroidism (Hunter, 2002).

Respiratory support included nebulized ipratropium/albuterol for bronchospasm (Venter et al., 2019), vasopressors for hemodynamic instability (Moura et al., 2016), and antibiotics for infection (Moura et al., 2016; Bustos Lozano et al., 1988). Gastrointestinal issues such as reflux and vomiting were managed with ranitidine and metoclopramide (Singh et al., 2013). Peri-anesthetic guidance emphasized airway vulnerability and careful extubation strategies (Hirakawa et al., 2017).

#### 4.5.2 Surgical interventions

Surgery is frequently required for progressive multisystem involvement. Spinal surgery is common: severe spondylolisthesis/scoliosis requiring L4-S1 fusion (Rojnueangnit et al., 2013); marked scoliosis corrected at 14 years (Schneider et al., 2013); earlier reports of resection of calcified ligamenta flava for cord compression (Miyazaki et al., 1990). Across cohorts, posterior spinal fusion and deformity correction predominate (Welborn et al., 2018; Labonne et al., 2016), with decompressive laminectomy for stenosis (Ishida et al., 1992; Morino et al., 2016; Hirakawa et al., 2017; Upadia et al., 2017). Severe deformities can cause tracheal compression, necessitating multidisciplinary care (Nishimoto et al., 2014). Cervical decompression/fusion has been reported for cord involvement (Hunter, 2002).

Cardiac procedures include mitral repair, tricuspid annuloplasty, and valve replacements (Wakami et al., 2022; Yoshida et al., 2015; Massin et al., 1999); vascular interventions such as balloon dilation/sympathectomy have been performed (Partington et al., 1988). Cryptorchidism repairs are frequent (Özden et al., 1994; Proud et al., 1992), alongside inguinal/umbilical hernia surgeries across ages (Manouvrier-Hanu et al., 1999; Horn et al., 2001; Lopez-Jimenez and Gimenez-Prats, 2003); hydrocele repair appears in several reports. Urological surgery for bilateral vesicoureteral reflux (Ronce et al., 1999) and a rare hysterectomy for uterine prolapse (Temtamy et al., 1975b) are described. ENT procedures include adenoidectomy/tonsillectomy, tympanostomy tubes, mastoidectomy for chronic otitis media (Quintela et al., 2015; Hartsfield et al., 1993; Hunter, 2002); tracheotomy for OSA/airway concerns (Hunter, 2002; Imataka et al., 2016); dental extractions under GA for aspiration prevention/oral care (Lopez-Jimenez and Gimenez-Prats, 2003; Norderyd and Aronsson, 2012). Additional operations include, cataract surgery (Dolman and Wright, 1978), vitreoretinal surgery for detachment (Singh et al., 2013), and hip dislocation repair (Proud et al., 1992). This breadth highlights the complex, progressive, systemic nature of CLS and the need for coordinated, multidisciplinary care.

### 4.6 Diagnostic interventions

#### 4.6.1 Genetic architecture and mutational hotspots

The RPS6KA3 variants identified in this review were distributed across all 22 coding exons, consistent with the absence of a single mutational hotspot and confirming the gene’s broad vulnerability to loss-of-function changes. Nevertheless, several residues including Arg259, Arg300, Arg310, and Arg729 appeared recurrently across unrelated cases, suggesting region-specific functional constraint within the N- and C-terminal kinase domains. All reported variants remain individually rare, with minor allele frequencies (MAF) below 0.0001 in large-scale population databases such as gnomAD and the 1000 Genomes Project. This extreme rarity, coupled with the high probability of loss-of-function intolerance (pLI = 1.0) for RPS6KA3, underscores its dosage sensitivity and explains why even single truncating or disruptive variants can produce the CLS.

#### 4.6.2 Diagnostic interventions, interpretation challenges, and AI-driven prioritization

Diagnostic confirmation of CLS has evolved substantially over the past decade. Earlier reports relied on targeted Sanger sequencing of *RPS6KA3*, whereas recent studies increasingly employ next-generation sequencing (NGS), whole-exome sequencing (WES), and chromosomal microarray analysis (CMA) to detect both sequence variants and copy-number changes. These methodological advances have improved detection of atypical presentations, including mosaic or mildly affected females. However, diagnostic challenges persist, particularly for intronic, synonymous, or splice-region variants that lack functional assays, as well as for females with skewed X-inactivation in whom the phenotype may be subtle or variable. In this context, AI-based variant prioritization tools such as CADD, REVEL, and AlphaMissense are emerging as valuable pre-screening resources that integrate conservation, structural, and population data. While such algorithms can enhance efficiency in variant ranking, their outputs must be interpreted cautiously and validated through expert clinical curation using ACMG–AMP criteria to ensure diagnostic accuracy.

#### 4.6.3 Variants of uncertain significance (VUS)

A small subset of RPS6KA3 variants identified in this review were classified as Variants of Uncertain Significance (VUS) under ACMG 2015 guidelines. These were predominantly missense substitutions lacking segregation or functional data, which limited definitive classification as pathogenic or likely pathogenic. One example includes the missense variant c.748G > A (p.Asp250Asn) reported by Tan et al. (2023), described as a VUS due to absence of prior reports and uncertain functional impact. Although VUS were retained for completeness, they were not incorporated into genotype phenotype correlation or frequency analyses to preserve analytical accuracy. Transparent reporting of such findings remains important in rare-disease genomics, as ongoing functional and familial studies may facilitate future reclassification and refine diagnostic interpretation in CLS.

Recent progress also includes the application of long-read sequencing for preimplantation genetic testing (PGT-M). Wen et al. (2023) demonstrated that Oxford Nanopore sequencing combined with the MARSALA platform allowed haplotype construction and accurate embryo diagnosis for a female carrying a *de novo* RPS6KA3 mutation. This approach avoided the need for additional family reference samples and led to a successful pregnancy with the birth of a healthy, unaffected child, highlighting its clinical feasibility for CLS and other monogenic disorders.

Complementing human genomic studies, translational insights from animal models have reinforced the central role of RPS6KA3 in neurodevelopment. RSK2 knockout mice and *Drosophila* models reproduce key phenotypic features of CLS, including impaired spatial learning, abnormal fear conditioning, and deficits in glutamatergic synaptic signaling (Fischer and Raabe, 2018). These findings underscore that disruption of RSK2 alters neuronal circuitry at a systems level, strengthening the rationale for comprehensive genomic testing in humans and informing potential future therapeutic strategies.

### 4.7 Germline mosaicism

Although CLS is typically caused by *de novo* variants, multiple reports have documented recurrence in families where mothers tested negative for the proband’s mutation using standard assays. Subsequent re-analysis with more sensitive techniques revealed maternal germline or low-level somatic mosaicism (Labonne et al., 2016; Giorgio et al., 2017). This carries important counseling implications: recurrence risk in ostensibly unaffected mothers is not negligible, and a negative blood test does not exclude transmission. Mosaicism underscores the value of high-sensitivity assays (deep sequencing, droplet digital PCR) when recurrence is suspected and should be explicitly discussed during genetic counseling.

### 4.8 Reproductive planning and PGT-A/M

Advances in assisted reproductive technology expand options for affected families. PGT-M permits selection of embryos without the familial RPS6KA3 variant during IVF. When combined with PGT-A, which screens for chromosomal aneuploidies that may reduce implantation success or cause miscarriage, families can adopt a dual-layered risk-reduction strategy. These technologies are particularly relevant where maternal germline mosaicism sustains recurrence risk despite negative parental testing. While access may be limited in some settings, integrating PGT-M (±PGT-A) into counseling reflects contemporary, preventive reproductive medicine and leverages the precise genotype phenotype data aggregated here.

### 4.9 Implications for genomic diagnosis and counseling

These findings carry direct implications for clinical diagnosis and genetic counseling, aligning with the broader goals of this review to inform both genomic practice and family decision-making. Beyond simple loss-of-function, the spectrum of RPS6KA3 variants demonstrates considerable mechanistic diversity, including missense changes at exon–intron boundaries that cause exon skipping, balanced X-autosome translocations with skewed X-inactivation leading to severe female phenotypes, and intragenic or whole-gene duplications that produce dosage-driven neuropsychiatric presentations with minimal dysmorphism. Such heterogeneity underscores that sequencing alone may not be sufficient: exon-level copy number variants (CNVs), structural rearrangements, and splicing defects may be overlooked without complementary methods such as chromosomal microarray (CMA), multiplex ligation-dependent probe amplification (MLPA), RNA studies, or, in selected cases, whole-genome sequencing (WGS).

From a counseling perspective, this complexity translates into equally nuanced challenges. Phenotypic variability within families and across mutation types makes prognosis uncertain and complicates recurrence risk estimation, particularly when germline mosaicism cannot be excluded. In reproductive planning, reliance solely on negative trio-WES in unaffected parents may create a false sense of reassurance. Hypothetical but realistic scenarios can arise where a couple proceeds with a second pregnancy under this assumption, only for invasive prenatal testing such as amniocentesis to later reveal the same pathogenic RPS6KA3 variant, with devastating emotional and ethical consequences. Stronger anticipatory guidance is therefore critical. Rather than waiting for mid-pregnancy confirmation, couples should be counseled about the option of preimplantation genetic testing for monogenic disorders (PGT-M), which provides earlier and more definitive reassurance while reducing the psychosocial burden of uncertainty.

Finally, the scope of counseling should extend beyond molecular recurrence risks to encompass the lived experience of affected individuals. Increasing recognition of neurobehavioral and psychiatric features in CLS highlights the importance of multidisciplinary counseling that includes not only medical prognosis but also educational, developmental, and psychosocial planning. Together, these considerations argue for a genomic-era standard of care in CLS comprehensive molecular diagnostics integrated with empathetic, forward-looking counseling that empowers families to make informed reproductive and clinical decisions.

### 4.10 Limitations and future directions

This review has several limitations, reflecting challenges inherent to rare disease synthesis. Most included studies were case reports or small series, risking publication bias toward unusual or severe presentations and underrepresenting milder or negative findings. Phenotypic detail was often incomplete, especially for psychiatric, endocrine, and longitudinal cardiopulmonary domains, potentially underestimating prevalence for features less routinely assessed. Genotype reporting varied in granularity; although we standardized variant classes, statistical power to resolve genotype–phenotype associations was limited by sample size and uneven class distribution. X-inactivation studies in females were not uniformly performed, constraining inference about its contribution to variability. Another limitation of this review is the heterogeneity in how age was reported across case studies. Some authors specified age at clinical presentation or diagnosis, while others listed current age at the time of report. Consequently, the mean and range of ages in this review represent the *reported age* rather than a standardized reference point. Although this introduces minor variability in descriptive statistics, it does not affect genotype phenotype interpretation or the qualitative synthesis of clinical features.

Future progress will depend on international registries with standardized, longitudinal clinical annotation and harmonized genomic data. Priorities include deep phenotyping (neurocognitive batteries, MRI), functional genomics (including RNA studies for splice-region variants), and systematic assessment of cardiac and respiratory trajectories. Embedding genetic-counseling frameworks that explicitly address germline mosaicism and reproductive options (PGT-M ± PGT-A) will translate discovery into meaningful, family-centered care.

## 5 Conclusion

This systematic review provides the most comprehensive synthesis to date of the phenotypic and genotypic spectrum of CLS in the molecular era. Developmental delay and intellectual disability remain near-universal, but systemic features including neurologic, musculoskeletal, cardiac, endocrine, and psychiatric manifestations are increasingly recognized and broaden the traditional triad. Genotype phenotype correlations show partial clustering of neurologic complications with truncating variants, yet overall severity is highly variable, reflecting X-inactivation, genetic modifiers, and stochastic developmental factors. The findings support multidisciplinary surveillance, early comprehensive genetic testing, and counseling that includes maternal germline mosaicism. Preventive reproductive options such as PGT-M, often combined with PGT-A, are emerging as meaningful strategies for at-risk families. By integrating quantitative prevalence estimates with detailed literature synthesis, this review serves as a resource not only for clinicians and researchers, but also for patients and families, supporting informed care, counseling, and future research in this rare yet impactful disorder.

## Data Availability

The original contributions presented in the study are included in the article/Supplementary Material, further inquiries can be directed to the corresponding author.
