# Supplementary material for: Coffin–Lowry syndrome: a systematic review of RPS6KA3 confirmed cases and implications for diagnosis and counseling
Source: Front Genet. 2026 Jan 12;16:1715229. doi: 10.3389/fgene.2025.1715229 (PMC12832115; doi:10.3389/fgene.2025.1715229)
Supplement: Supplementary file 1 [file Table1.docx]

Supplementary Material

**Supplementary Table 1. Phenotypic characteristics of 72 genetically confirmed Coffin–Lowry syndrome cases.**
Individual case-level data are presented, including demographic details (author/year, country, gender, and age at diagnosis), and systematically extracted clinical features. Phenotypic domains include developmental delay, intellectual disability (graded as mild, moderate, severe, or profound), neurobehavioral/psychiatric manifestations (including autism), neurologic features (stimulus-induced drop attacks, spasticity/paraplegia, seizures), musculoskeletal anomalies (kyphoscoliosis/other spine deformity, pectus carinatum/excavatum), cardiovascular involvement (cardiomyopathy, valvular abnormalities), and additional systemic features (dental anomalies, hearing loss, vision impairment). Cases where information was not reported are indicated as “Unknown.”

| **Case ID** | **Author, Year** | **Country** | **Gender** | **Age (years or months)** | **Developmental Delay** | **Intellectual Disability (Mild / Moderate / Severe / Profound)** | **Neurobehavioral/Psychiatric (include Autism)** | **Neurologic – Stimulus-Induced Drop Attacks** | **Neurologic – Spasticity/Paraplegia** | **Neurologic – Seizures** | **Musculoskeletal – Kyphoscoliosis/Other Spine Deformity** | **Musculoskeletal – Pectus Carinatum/Excavatum** | **Cardiovascular – Cardiomyopathy** | **Cardiovascular – Valvular Abnormalities** | **Dental Issues / Loss of Teeth** | **Hearing Loss** | **Vision Issues** |
| --- | --- | --- | --- | --- | --- | --- | --- | --- | --- | --- | --- | --- | --- | --- | --- | --- | --- |
| CLS001 | Pantani et al. 2025 | Italy | Female | 5 years | Yes | Not Reported | Not Reported | Yes | No | Yes | No | Yes | No | No | No | Not Reported | Not Reported |
| CLS002 | Kim et al. 2025 | USA | Female | 15 years | Yes (mild motor & speech delay) | Mild | Psychosis, ADHD, anxiety, obsessive-compulsive traits | No | No | No | No | No | No | No | Yes (mottled dentition) | Yes (history of otitis media) | Yes (amblyopia) |
| CLS003 | Tan et al. 2023 | Malaysia | Male | 3 years (diagnosed at ~1 year) | Yes | Not Reported | Not Reported | Not Reported | Not Reported | Not Reported | Not Reported | Yes | No | No | Not Reported | No | Not Reported |
| CLS004 | Song et al. 2023 | Korea | Female | 5 years | Yes | Not Reported | Not Reported | Yes | No | No | Yes (scoliosis 23° Cobb) | Yes | No | No | Yes (malocclusion, hypodontia) | No | Yes (astigmatism) |
| CLS005 | Kocaaga & Yimenicioglu 2023 | Turkey | Male | 4 years | Yes | Moderate | Not Reported | Not Reported | No | No | No | No | No | No | Yes (widely spaced teeth) | No | No |
| CLS006 | Tise et al. 2022 | USA | Female | 30 months | Yes | Not Reported | Not Reported | No | No | No | No | No | No | No | No | No | No |
| CLS007 | Jin et al. 2022 | China | Male | 5 months | Yes | Not reported | Not reported | No | No | No | No | No | No | Yes – persistent left superior vena cava | No | Yes | No |
| CLS008 | Jin et al. 2022 | China | Male | 5 months | Yes | Not reported | Not reported | No | No | No | No | No | No | Yes – mild tricuspid regurgitation | No | Yes | No |
| CLS009 | Gürsoy et al. 2022 | Turkey | Female | 7.5 years | Yes | Mild | Compulsive eyebrow-pulling behavior | No | No | No | Yes | No | No | No | No | No | No |
| CLS010 | Gürsoy et al. 2022 | Turkey | Male | 15 months | Yes | Not Reported | Not Reported | No | No | Yes (focal tonic-clonic) | No | No | No | No | No | No | No |
| CLS011 | Cong et al. 2022 | China | Female | 17 years | Yes | Moderate | Not Reported | No | No | No | No | No | No | Yes (mild MR & TR) | No | No | No |
| CLS012 | Cong et al. 2022 | China | Female | 21 years | Yes | Severe | Not Reported | No | No | No | No | No | No | Yes (mild MR & TR) | No | No | No |
| CLS013 | Cong et al. 2022 | China | Female | 47 years | Yes | Severe | Not Reported | No | No | No | Yes (scoliosis) | No | No | Yes (mild MR & TR) | No | No | No |
| CLS014 | Boulos et al. 2021 | Lebanon | Male | ~36 years (deceased) | Yes | Mild | Not Reported | Not Reported | No | No | No | No | Yes (congestive heart disease) | No | No | No | No |
| CLS015 | Boulos et al. 2021 | Lebanon | Male | 38 years | Yes | Not Reported | Paroxysmal hand movements | Not Reported | No | No | No | No | No | Mild sclerosis mitral & aortic valves; minimal MR & TR | No | No | No |
| CLS016 | Boulos et al. 2021 | Lebanon | Female | ~38 years | No | No | Not Reported | No | No | No | No | No | No | No | No | No | No |
| CLS017 | Boulos et al. 2021 | Lebanon | Male | 4 years | Yes | Not Reported | Not Reported | No | No | No | No | No | No | No | No | No | No |
| CLS018 | Boulos et al. 2021 | Lebanon | Male | 7 years | No | No | Not Reported | No | No | No | Yes (minimal scoliosis) | No | No | No | No | No | No |
| CLS019 | Boulos et al. 2021 | Lebanon | Female | 3 years | No | No | Not Reported | No | No | No | No | No | No | No | No | No | No |
| CLS020 | Di Stazio et al. 2021 | Italy | Male | 20 years | Yes | Severe (mild at 13y → severe at 18y) | ADHD, hyperactivity, impulsiveness/aggressiveness | No | No | No | Yes (scoliosis) | No | No | No | No | No | Yes (right amblyopia) |
| CLS021 | Di Stazio et al. 2021 | Italy | Male | 48 years | Yes | Mild–Moderate | Behavioral problems, social interaction difficulties, stress intolerance | No | No | No | No | No | No | No | No | No | Yes (strabismus, amblyopia) |
| CLS022 | Yamoto et al. 2020 | Japan | Female | 6 years | Yes | Severe | Not Reported | No | No | Yes (myoclonic, infancy-early childhood, resolved by 5y) | No | No | No | No | Yes (hypodontia, widely spaced teeth, large medial incisors) | No | Yes (myopia) |
| CLS023 | Uliana et al. 2019 | Italy | Male | 34 years | Not Reported | Mild-Moderate | Paranoid schizophrenia | No | No | No | No | No | No | No | No | No | No |
| CLS024 | Uliana et al. 2019 | Italy | Male | 63 years | Not Reported | Mild-Moderate | Psychosis | No | No | Yes (single seizure at 25y) | No | No | No | No | No | No | No |
| CLS025 | Uliana et al. 2019 | Italy | Female | 59 years | No | No | Major depression | No | No | No | No | No | No | No | No | No | No |
| CLS026 | Lv et al. 2019 | China | Male | 12 months | Yes | Severe | Not Reported | No | No | No | No | No | No | No | No | Yes (>85 dB both ears |  |
| CLS027 | Fung et al. 2019 | Hong Kong (China) | Male | 10 years | Yes | Moderate-Severe | Not Reported | No | No | No | Yes (mild kyphoscoliosis) | No | No | ASD (small) | No | No | No |
| CLS028 | Fung et al. 2019 | Hong Kong (China) | Female | 36 years | No | Nil (high school) | Not Reported | No | No | No | Yes (mild kyphoscoliosis) | No | No | No | No | No | No |
| CLS029 | Fung et al. 2019 | Hong Kong (China) | Male | 3 years | Yes | N/A | Not Reported | No | No | No | Yes (mild kyphoscoliosis) | No | No | No | No | Yes (bilateral sensorineural) | No |
| CLS030 | Fung et al. 2019 | Hong Kong (China) | Female | 19 years | Yes | Moderate | Not Reported | No | No | No | No | No | No | No | No | No | No |
| CLS031 | Fung et al. 2019 | Hong Kong (China) | Female | 42 years | Yes | Moderate | Not Reported | No | No | No | No | No | No | No | No | No | No |
| CLS032 | Fung et al. 2019 | Hong Kong (China) | Male | 2 years | Yes | Moderate | Not Reported | No | No | No | No | No | No | No | No | No | No |
| CLS033 | Fung et al. 2019 | Hong Kong (China) | Male | 18 years | Yes | Mild (special school) | Not Reported | No | No | No | Yes (kyphoscoliosis) | No | No | No | No | No | No |
| CLS034 | Fung et al. 2019 | Hong Kong (China) | Female | 16 years | Yes | Mild | Not Reported | No | No | No | Yes (kyphoscoliosis) | No | No | No | No | No | Yes (amblyopia) |
| CLS035 | Fung et al. 2019 | Hong Kong (China) | male | 25 years | Yes | Mild (special school) | Not Reported | No | No | No | Yes (rapid scoliosis progression) | No | No | No | No | No | no |
| CLS036 | Castelluccio et al. 2019 | USA | Male | 12 years | Yes | Moderate | Not Reported | No | No | Yes (febrile seizure, single episode) | Yes (moderate neuromuscular scoliosis, syrinx T6-T12) | Yes (pectus carinatum) | No | No | Yes (hypodontia, bifid uvula) | Possible conductive | No |
| CLS037 | Castelluccio et al. 2019 | USA | Male | 11 years | Yes | Moderate | Not Reported | No | No | No | Yes (minimal levoscoliosis) | Yes (pectus carinatum) | No | No | Yes (hypodontia, widely spaced teeth) | No | Pseudo-esotropia |
| CLS038 | Castelluccio et al. 2019 | USA | Male | 9 years | Yes | Moderate | Not Reported | Yes | No | No | Yes (moderate neuromuscular scoliosis) | Yes (pectus carinatum) | No | No | Yes (hypodontia) | No | No |
| CLS039 | Brás et al. 2019 | Portugal | Female | 21 years | Yes | Severe | Not Reported | Yes (drop episodes, not stimulus-induced) | No | Yes (tonic nocturnal seizures) | No | No | No | No | No | No | No |
| CLS040 | Nikfar et al. 2018 | Iran | Male | 14 years | Yes | Mild | Not Reported | No | No | Yes | No | No | No | No | Yes (hypodontia) | No | No |
| CLS041 | Miyata et al. 2018 | Japan | Male | 2 years | Yes | Severe | Not Reported | No | No | No | No | No | No | No | Yes (widely spaced teeth) | No | No |
| CLS042 | Upadia et al. 2017 | USA | Male | 3 months (presentation) | Yes | Mild | Not Reported | No | No | No | No | No | No | No | No | No | No |
| CLS043 | Labonne et al. 2016 | USA | Male | 14 years | Yes | Severe | Not Reported | No | psychomotor delays and  athetotic movements | No | Kyphoscoliosis (progressive, 70°) | No | No | VSD (small, perimembranous) | Teeth fall out at root, delayed regrowth | Yes (bilateral) | Yes (hyperopia) |
| CLS044 | Tos et al. 2015 | Turkey | Male | 6 years | Yes | Severe | Not Reported | No | Spasticity (upper limbs), Hypotonia (lower limbs) | Febrile seizure at 1 mo | Hip dislocation | Yes (pectus carinatum) | No | No | No | No | No |
| CLS045 | Tos et al. 2015 | Turkey | Male | 3 years | Yes | Severe | Not Reported | No | Spasticity (upper & lower limbs) | No | No | Yes (pectus carinatum) | No | No | No | No | No |
| CLS046 | Tos et al. 2015 | Turkey | Male | 12 years | Yes | Mild | Not Reported | No | No | No | No | No | No | No | No | No | No |
| CLS047 | Bertini et al. 2015 | Italy | Male | 12 years | Yes | Mild | Not Reported | No | No | No | No | No | No | No | Yes (dental crowding, high palate) | Yes (moderate bilateral sensorineural) | No |
| CLS048 | Rojnueangnit et al. 2014 | USA | Female | 12 years | Yes | Severe | Not Reported | Yes | No | No | Yes (progressive scoliosis, spondylolisthesis) | Yes (pectus excavatum) | No | No | Yes (widely spaced teeth) | No | No |
| CLS049 | Nishimoto et al. 2014 | USA | Male | Not reported | Yes | Severe | Not Reported | Yes | Yes (lordosis, severe kyphoscoliosis) | No | Yes (severe kyphoscoliosis + lordosis, rib deformities) | Yes (small chest cavity) | No | Mild mitral regurgitation | Loss of two upper medial incisors | No | No |
| CLS050 | Nishimoto et al. 2014 | USA | Male | 3 years | Yes | Severe | Not Reported | Yes | No | Yes (single grand mal at 3 y) | Yes (severe scoliosis) | Yes (pectus carinatum) | No | No | Peg-shaped incisors | No | Right esotropia |
| CLS051 | Nishimoto et al. 2014 | USA | Male | 10 years | Yes | Severe | ADHD | No | No | No | Yes (scoliosis) | Yes (pectus excavatum) | No | No | Loss of incisors | No | No |
| CLS052 | Loupe et al. 2014 | USA | Female | 4.5 months | Yes | Not Reported | Not Reported | No | No | No | No | No | No | No | No | No | No |
| CLS053 | Loupe et al. 2014 | USA | Female | 9 years | Yes | Not Reported | Not Reported | No | No | No | No | No | No | No | No | No | No |
| CLS054 | Loupe et al. 2014 | USA | Female | 7 years | Yes | Not Reported | Not Reported | No | No | No | No | No | No | No | No | No | No |
| CLS055 | Loupe et al. 2014 | USA | Female | 4 years | Yes | Not Reported | Not Reported | No | No | No | No | No | No | No | No | No | No |
| CLS056 | Arslan et al. 2014 | Turkey | Female | 10years | Yes | Moderate | Not Reported | Yes | No | No | Yes (kyphosis) | Yes (pectus excavatum) | No | No | No | No | No |
| CLS057 | Matsumoto et al. 2013 | Japan | Male | 15 years | Yes | Mild | ADHD, panic attacks | No | No | Yes (localization-related epilepsy, onset 13 y) | No | No | No | No | No | No | No |
| CLS058 | Matsumoto et al. 2013 | Japan | Male | 13 years | Yes | Mild | Not Reported | No | No | Yes ( complex partial seizure, onset 10 y) | No | No | No | No | No | No | No |
| CLS059 | Matsumoto et al. 2013 | Japan | Female | 6 years | Yes | Not Reported | Pervasive Developmental Disorder | No | No | No | No | No | No | No | No | No | No |
| CLS060 | Matsumoto et al. 2013 | Japan | Male | 4 years | Yes | Borderline | ADHD | No | No | No | No | No | No | No | No | No | No |
| CLS061 | Tejada et al. 2011 | Spain | Male | 8 years | Yes | Mild | ADHD, attention deficit, mild motor hyperactivity | No | No | No | No | No | No | No | No | No | No |
| CLS062 | Tejada et al. 2011 | Spain | Male | 5 years | Yes | Mild | Not Reported | No | No | No | No | No | No | No | No | No | No |
| CLS063 | Tejada et al. 2011 | Spain | Male | 36 years | Yes (childhood) | Not Reported | Not Reported | No | No | No | No | No | No | No | No | No | No |
| CLS064 | Tejada et al. 2011 | Spain | Male | 22 years | Yes (childhood) | Not Reported | Not Reported | No | No | No | No | No | No | No | No | No | No |
| CLS065 | Tejada et al., 2011 | Spain | Female | 27 Years | No | No | No | No | No | No | No | No | No | No | No | No | No |
| CLS066 | Tejada et al., 2011 | Spain | Female | Not reported | No | No | No | No | No | No | No | No | No | No | No | No | No |
| CLS067 | Senel et al. 2011 | Turkey | Male | 6 years | Yes | Severe | Not Reported | No | No | No | Spina bifida (L4-L5, S1), cleft of C4 vertebra | No | No | No | Yes (oligodontia) | No | Yes (optic atrophy) |
| CLS068 | Martinez et al. 2011 | USA | Male | 13 years | Yes | Severe | Not Reported | No | Left-sided weakness post-cardiac arrest | No | No | No | Yes (LV noncompaction with restrictive cardiomyopathy) | Yes (mitral valve prolapse, regurgitation) | No | No | No |
| CLS069 | Jurkiewicz et al. 2010 | Poland | Female | 2 years | Yes | Moderate | Hyperactivity, anxiety | No | No | No | Kyphosis, scoliosis | Yes (pectus carinatum) | No | No | Yes (hypodontia, neonatal tooth) | No | No |
| CLS070 | Jurkiewicz et al. 2010 | Poland | Female | 5 years | Yes | Moderate | Not Reported | No | No | No | Scoliosis | No | No | No | Yes (hypodontia) | No | No |
| CLS071 | Jurkiewicz et al. 2010 | Poland | Female | 6 months | Yes | Mild | Not Reported | No | No | No | No | No | No | No | No | No | No |
| CLS072 | Jurkiewicz et al. 2010 | Poland | Female | 19 years | Yes | Mild | Not Reported | Yes | No | No | No | No | No | No | No | No | No |

**Supplementary Table 2. Genotypic spectrum of RPS6KA3 variants in genetically confirmed Coffin–Lowry syndrome cases.**
Case-level molecular data are summarized, including mutation type (frameshift, missense, nonsense, splice-site, duplication, deletion, structural variant, or other), zygosity (hemizygous, heterozygous, or mosaic), ACMG classification (pathogenic, likely pathogenic, or variant of uncertain significance), copy-number variant (CNV) detection status, and approximate variant location (exon, intron/splice region, multi-exon CNV, or gene-level event). Where available, additional details such as exon/intron coordinates, functional effects (e.g., exon skipping, truncating), and structural rearrangements are included. Variants not reported in sufficient detail are indicated as “Not specified.”

| **Author, Year** | **Country** | **Exon/Intron** | **cDNA Change** | **Protein Change** | **Mutation Type** | **Zygosity** | **Diagnostic Method** | **ACMG Classification** | **CNV Detected?** | **Brief Phenotype Summary** | **Notes** |
| --- | --- | --- | --- | --- | --- | --- | --- | --- | --- | --- | --- |
| Pantani et al., 2025 | Italy | Exon 20 | c.1954C>T (p.Arg652*) | Leu467 (missense) | Missense | Heterozygous | Exome sequencing | Pathogenic (PVS1 + PM2 + PP4) | No | Myoclonic epilepsy, SIDEs, dysmorphism, ID | Variant at Leu467, not exact cDNA given, familial segregation, in silico damaging |
| Ji-Sun Kim, 2025 | Korea | Intron 17 | c.1602 + 2del | Not specified | Splice-site | Heterozygous (de novo) | Targeted RPS6KA3 sequencing + del/dup analysis | Likely Pathogenic (PVS1 + PM2 + PS2 de novo + PP4) | No | Tapered fingers, classic CLS features | Splice donor site variant, not in gnomAD, parental de novo |
| Tan, 2023 | Not specified | Exon 9 | c.748G>A | p.Asp250Asn | Missense | Hemizygous | Whole exome sequencing | VUS (PM2 + PP4 only) | No | CLS, intellectual disability | KCNQ1, DNAH9, SP110, SPATA7 also detected; only RPS6KA3 relevant for CLS |
| Song, 2021 | Korea | Exon 3 | c.326_338delinsCTCGAGAC | p.Val109Alafs*10 | Frameshift | Heterozygous (de novo) | Targeted exome sequencing (xGen panel) + Sanger validation | Likely Pathogenic (PVS1 + PS2 + PM2 + PP4) | No | CLS features | Novel variant, not in gnomAD/parents/controls, truncating, diagnosed as CLS |
| Kocaaga, 2023 | Turkey | Exon 22 | c.2186G>A | p.Arg729Gln | Missense | Hemizygous | Whole-exome sequencing + Sanger validation | Pathogenic (PS1 + PM1 + PM2 + PP4) | No | CLS features | Mother heterozygous carrier; SIFT, PolyPhen-2, Mutation Taster all predict pathogenicity |
| Tise, 2022 | Not specified | Intron 4 | c.325+1G>T | Not specified | Splice-site | Heterozygous (maternally inherited) | Exome sequencing | Likely pathogenic | No | CLS, phenotypic variation mother/proband | Canonical splice site, null allele; X-inactivation: complete skew in mother, random in proband; de novo in mother |
| Huiying Jin, 2022 | China | Exon 11 | c.898C>T | p.R300* | Nonsense | Hemizygous | Whole-exome sequencing + Sanger validation | Pathogenic (PVS1 + PS2 + PM2 + PP4) | No | CLS features | De novo mutation, twins |
| Huiying Jin, 2022 | China | Exon 11 | c.898C>T | p.R300* | Nonsense | Hemizygous | Whole-exome sequencing + Sanger validation | Pathogenic (PVS1 + PS2 + PM2 + PP4) | No | CLS features | De novo mutation, twins |
| Semra Gürsoy, 2022 | Turkey | Exon 7 | c.593A>T | p.N198I | Missense | Heterozygous | Targeted X-linked ID panel (Celemix) + Sanger | Likely Pathogenic (PM1 + PM2 + PP2 + PP3) | No | CLS, compulsive eyebrow-pulling | Novel, not in literature, ACMG: PM1, PM2, PP2, PP3; wild-type in parents/sibling |
| Semra Gürsoy, 2022 | Turkey | Exon 3 | c.161dup | p.His54GlnfsTer8 | Frameshift | Hemizygous | Targeted X-linked ID panel (Celemix) + Sanger | Pathogenic (PVS1 + PM1 + PM2 + PP2 + PP3 + PM6) | No | CLS | Novel, not in literature, ACMG: PVS1, PM1, PM2, PP2, PP3, PM6; wild-type in parents |
| Yan Cong, 2022 | China | Exon 11 | c.898C>T | p.R300X | Nonsense | Heterozygous | WES + Sanger sequencing + CNV-seq | Pathogenic (PVS1, PM2, PP1, PP3, PP4, PP5) | No (for RPS6KA3); Yes (distal 22q11.2 del) | CLS features | Marked XCI skewing, qPCR mRNA expression higher than relatives |
| Yan Cong, 2022 | China | Exon 11 | c.898C>T | p.R300X | Nonsense | Heterozygous | WES + Sanger sequencing + CNV-seq | Pathogenic (PVS1, PM2, PP1, PP3, PP4, PP5) | Yes (distal 22q11.2 del) | CLS features | XCI random, qPCR mRNA expression lower than proband, CNV 22q11.2 microdeletion |
| Yan Cong, 2022 | China | Exon 11 | c.898C>T | p.R300X | Nonsense | Heterozygous | WES + Sanger sequencing + CNV-seq | Pathogenic (PVS1, PM2, PP1, PP3, PP4, PP5) | Yes (distal 22q11.2 del) | CLS features | XCI random, qPCR mRNA expression lower than proband, CNV 22q11.2 microdeletion |
| Boulos et al., 2021 | Lebanon | Exon 19 | c.1784A>G | p.Tyr595Cys | Missense | Hemizygous (male) | WES + Sanger sequencing | Likely pathogenic | No | Male (III-5): mild ID, craniofacial dysmorphism, tapered fingers, fullness of forearms, type 2 diabetes at 30y, adrenal gland tumor at 36y, congestive heart disease, pulmonary embolism | Died at 36y; no known diabetes risk factors |
| Boulos et al., 2021 | Lebanon | Exon 19 | c.1784A>G | p.Tyr595Cys | Missense | Hemizygous (male) | WES + Sanger sequencing | Likely pathogenic | No | Male (III-6): cognitive impairment, paroxysmal hand movements, fullness of forearms, craniofacial dysmorphism, cardiac abnormalities (LV hypokinesia, mild sclerosis of mitral/aortic valves, minimal MR/TR insufficiency), type 2 diabetes at 36y | No diabetes risk factors |
| Boulos et al., 2021 | Lebanon | Exon 19 | c.1784A>G | p.Tyr595Cys | Missense | Heterozygous (female) | WES + Sanger sequencing | Likely pathogenic | No | Female (II-5, mother of III-5/III-6): normal intelligence, mild facial/hand features, type 2 diabetes at 38y | No diabetes risk factors |
| Boulos et al., 2021 | Lebanon | Exon 19 | c.1784A>G | p.Tyr595Cys | Missense | Hemizygous (male) | PCR + sequencing of exon 19 | Likely pathogenic | No | Male (IV-1, 4y): developmental delay, fullness of forearms, typical craniofacial dysmorphism | BMI 16.6 kg/m² |
| Boulos et al., 2021 | Lebanon | Exon 19 | c.1784A>G | p.Tyr595Cys | Missense | Hemizygous (male) | PCR + sequencing of exon 19 | Likely pathogenic | No | Male (IV-2, 7y): minimal scoliosis, fullness of forearms, craniofacial dysmorphism, normal development | BMI 15.1 kg/m² |
| Boulos et al., 2021 | Lebanon | Exon 19 | c.1784A>G | p.Tyr595Cys | Missense | Heterozygous (female) | PCR + sequencing of exon 19 | Likely pathogenic | No | Female (IV-4, 3y): dysmorphic features (full lips, broad nose), normal growth and development | — |
| Di Stazio et al., 2021 | Italy | Exon 7 | c.566T>C | p.I189T | Missense | Hemizygous | NGS panel + segregation | Predicted pathogenic | No | Mild/atypical CLS, hypotonia, cognitive defect, dysmorphism, behavioral anomalies, macrocephaly | Highly conserved residue, not in gnomAD, X-inactivation skewed in mother, segregates in family |
| Di Stazio et al., 2021 | Italy | Exon 7 | c.566T>C | p.I189T | Missense | Hemizygous | NGS panel + segregation | Predicted pathogenic | No | Mild/atypical CLS, hypotonia, cognitive defect, dysmorphism, behavioral anomalies, macrocephaly | Maternal uncle, same features as above |
| Yamoto et al., 2020 | Japan | Intron 1 | None (fusion) | None | Structural variant (translocation, chrX;chr11) | Heterozygous | Karyotyping, aCGH, WGS, PCR | Not specified | No | CLS, female with balanced X;11 translocation | De novo translocation; fusion in intron 1 RPS6KA3; 6bp deletion and 4bp insertion; skewed X-inactivation |
| Castelluccio et al., 2019 | USA | Exons 5–9 | — (intragenic tandem duplication; breakpoints not resolved) | p.M261Tfs13* | Frameshift (tandem duplication) | Hemizygous (male) | WES + targeted high-resolution aCGH + qRT-PCR + cDNA analysis | Likely pathogenic | Yes — duplication of exons 5–9 in RPS6KA3 | Male (III-1, 12y): moderate ID, GDD, severe speech delay, short stature, microcephaly, submucous cleft palate, laryngomalacia, vesicoureteral reflux, dental anomalies, bifid uvula, pectus carinatum, scoliosis, thoracolumbar syrinx, supernumerary nipples, conductive hearing loss, mild OSA | Most severely affected of 3 brothers; no cardiovascular disease |
| Castelluccio et al., 2019 | USA | Exons 5–9 | — (intragenic tandem duplication; breakpoints not resolved) | p.M261Tfs13* | Frameshift (tandem duplication) | Hemizygous (male) | WES + targeted high-resolution aCGH + qRT-PCR + cDNA analysis | Likely pathogenic | Yes — duplication of exons 5–9 in RPS6KA3 | Male (III-2, 11y): moderate ID, GDD, short stature, dental anomalies, pectus carinatum, supernumerary & inverted nipples, broad-tapered fingers, exercise intolerance, pseudo-esotropia | Milder cognitive impairment than III-1 |
| Castelluccio et al., 2019 | USA | Exons 5–9 | — (intragenic tandem duplication; breakpoints not resolved) | p.M261Tfs13* | Frameshift (tandem duplication) | Hemizygous (male) | WES + targeted high-resolution aCGH + qRT-PCR + cDNA analysis | Likely pathogenic | Yes — duplication of exons 5–9 in RPS6KA3 | Male (III-3, 9y): moderate ID, GDD, short stature, dental anomalies, pectus carinatum, scoliosis, broad-tapered fingers, SIDEs | Cognitively more advanced than brothers; speaks short sentences |
| Uliana et al., 2019 | Italy | Multi-exon duplication | — (CNV; not applicable) | — (not applicable, gene-level duplication) | Microduplication | Hemizygous (male) | Array-CGH + qPCR | Likely pathogenic | Yes — arr[GRCh37] Xp22.12(19651494_20597642)x2 | Male (III-1, 34y): mild-moderate ID, paranoid schizophrenia, tall stature (187 cm), overweight, no dysmorphic or skeletal anomalies | RPS6KA3 mRNA > 2× normal male control; gene dosage effect |
| Uliana et al., 2019 | Italy | Multi-exon duplication | — (CNV; not applicable) | — (not applicable, gene-level duplication) | Microduplication | Hemizygous (male) | Array-CGH | Likely pathogenic | Yes — arr[GRCh37] Xp22.12(19651494_20597642)x2 | Male (II-3, 63y): mild-moderate ID, psychosis, epilepsy (single episode at ~25y) | — |
| Uliana et al., 2019 | Italy | Multi-exon duplication | — (CNV; not applicable) | — (not applicable, gene-level duplication) | Microduplication | Heterozygous (female) | Array-CGH + qPCR | Likely pathogenic | Yes — arr[GRCh37] Xp22.12(19651494_20597642)x2 | Female (II-2, 59y): major depression, normal cognition | RPS6KA3 mRNA ~0.62× normal control |
| Lv et al., 2019 | China | Exon 22 | c.2185C>T | p.Arg729Trp | Missense | Hemizygous (de novo) | NGS (Agilent Human Genome Panel) + Sanger | Not specified | No | CLS, growth concerns | De novo mutation; not present in mother or brother; RSK2 instability mentioned |
| Fung et al., 2019 | China | Exons 9-10 (deletion) | Deletion exons 9–10 | Not specified | Exonic deletion | Hemizygous | Targeted testing + RT-PCR/Sanger | Not specified | Yes | Severe GDD, dysmorphism, ASD, kyphoscoliosis | Maternal inheritance; Novel mutation |
| Fung et al., 2019 | China | Exons 9-10 (deletion) | Deletion exons 9–10 | Not specified | Exonic deletion | Heterozygous | Targeted testing + RT-PCR/Sanger | Not specified | Yes | Similar features, mild ID | Maternal inheritance; Novel mutation |
| Fung et al., 2019 | China | Exon 17 | c.1449T>A | p.Tyr483Ter | Nonsense | Hemizygous | Targeted testing + Sanger | Pathogenic | No | Typical CLS, dysmorphism, hearing loss, kyphoscoliosis | Maternal inheritance; Novel mutation |
| Fung et al., 2019 | China | Exon 17 | c.1449T>A | p.Tyr483Ter | Nonsense | Heterozygous | Targeted testing + Sanger | Pathogenic | No | Moderate ID, resembling facial features | Maternal inheritance; Novel mutation |
| Fung et al., 2019 | China | Exon 17 | c.1449T>A | p.Tyr483Ter | Nonsense | Heterozygous | Targeted testing + Sanger | Pathogenic | No | Moderate ID, resembling facial features | Maternal inheritance; Novel mutation |
| Fung et al., 2019 | China | Intron 19 (splice site) | c.1842-1G>T | Not specified | Splice-site | Hemizygous | Targeted testing + Sanger | Pathogenic | No | GDD, hypotonia, dysmorphism | Maternal inheritance; Novel mutation |
| Fung et al., 2019 | China | Exon 16 | c.1428_1430delTAT | Nonframeshift deletion | In-frame deletion | Hemizygous | Sequencing analysis | Pathogenic | No | Dysmorphism, scoliosis, short stature | De novo; Known variant (HGMD) |
| Fung et al., 2019 | China | Exon 9 | c.638G>A | p.Gly213Asp | Missense | Hemizygous | Targeted sequencing | Pathogenic | No | Macrocephaly, ID, hypotonia, hernia | De novo; Novel mutation |
| Fung et al., 2019 | China | Exon 7 | c.501delA | p.Glu168LysfsTer14 | Frameshift | Heterozygous | Sequencing analysis | Pathogenic | No | Mild ID, ADHD, scoliosis, amblyopia | De novo; Novel mutation |
| Brás et al., 2019 | Portugal | Not specified | c.1756dup | p.Ala586Glyfs*11 | Frameshift | Heterozygous (de novo) | Exome sequencing (TruSight One, Illumina) | Probably pathogenic | No | Bizarre gait, CLS diagnosis | De novo; Novel mutation; Not in ExAC, ESP, or dbSNP; Predicted NMD/truncated protein; Not present in parents. |
| Nikfar et al., 2018 | Iran | Exon 22 | c.2185C>T | p.Arg729Trp | Missense | Hemizygous (de novo) | WES + Sanger sequencing | Not specified | No | Mild MR, seizures, CLS features | De novo; Not present in either parent (both genetically normal); First molecular CLS case in Iran |
| Miyata et al., 2018 | Japan | Exon 18 | c.1682A>G | p.Asp561Gly | Missense | Hemizygous (de novo) | WES + Sanger sequencing | Likely Pathogenic (PM1 + PM2 + PP3 + PP4) | No | CLS, periventricular cystic brain lesions | De novo; Novel missense; Not present in parents; Supported by prediction software |
| Upadia et al., 2017 | USA | Not specified | c.328C>T | Not specified | Not specified | Hemizygous (de novo) | WES | Pathogenic | No | CLS, foramen magnum compression | De novo; Not present in parents; Consistent with CLS diagnosis |
| Tos et al., 2015 | Turkey | Exon 11 | c.898C>T | p.Arg300* | Nonsense | Hemizygous (presumed) | PCR + Sanger sequencing | Not specified | No | Severe MR, microcephaly, dysmorphism, MRI/CT abn. | Proband in large family; multiple affected siblings, only proband tested |
| Bertini et al., 2015 | Italy | Exons 1–7 (Xp22.12) | Not specified | Not specified | Partial in-tandem duplication | Hemizygous (male) | Array CGH, qRT-PCR, FISH, Copy Number Assay | Not classified (CNV) | Yes (partial duplication) | Mild intellectual disability; no classic CLS facial/skeletal features | Duplicated region: chrX:20,208,171–20,833,589 (hg19); confirmed in proband, mother, maternal uncle. Probes validated: intron 2 (duplicated), exon 21 (not duplicated). FISH: in-tandem duplication, not ectopic. qRT-PCR: reduced RPS6KA3 expression in proband and mother. Mother carrier, no classic phenotype, random X inactivation. Gene dosage effect likely; affects N-terminal kinase catalytic domain. |
| Rojnueangnit et al., 2014 | USA | Exon 17 | c.1570dupA | p.Thr524Asnfs*2 | Frameshift, truncating | Heterozygous (female) | Sanger sequencing (all exons), XCI analysis | Pathogenic (novel, truncating) | No | Classic CLS, female with drop episodes | Novel, de novo 1-bp duplication; not present in mother; causes frameshift at codon 524, premature stop at 525; likely no protein or truncated/nonfunctional protein due to NMD; random X-inactivation (71:29); N-terminal kinase domain preserved. |
| Nishimoto et al., 2014 | USA | Exons 15 & 16 | r.1228_1443del216; 4kb del chrX:20,187,287–20,191,368 | p.Q410_D481del (72aa del) | In-frame deletion | Hemizygous (male) | Microarray, transcript sequencing | Pathogenic (PVS1 + PM1 + PM2 + PP4) | Yes | Classic CLS, drop episodes | Historic family; novel 216bp deletion removes C-terminal kinase domain; loss-of-function. |
| Nishimoto et al., 2014 | USA | Intron 17 (splice-site mutation → exon 17 skipping) | c.1602 + 3_1602 + 6delAAGT (intron 17) | p.V482_G534del (53aa del) | Splice site; exon skip | Hemizygous (male) | mRNA/gDNA sequencing | Pathogenic (PVS1 + PM2 + PP4 + PS2)) | No | CLS, C-terminal KD loss | 4bp del in intron 17, exon 17 skipping; de novo; partial C-terminal KD deletion. |
| Nishimoto et al., 2014 | USA | Exon 4 | c.262dupA | p.Ile88Asnfs*5 | Frameshift, truncating | Hemizygous (male) | Sanger sequencing | Pathogenic (PVS1 + PM2 + PP4) | No | Severe, classic CLS | 1bp duplication; frameshift; reported previously (Zeniou et al., 2002; Jurkiewicz et al., 2010); truncated N-terminal KD only. |
| Loupe et al., 2014 | USA | Exon 9 | c.865_866delCA | p.Gln289ValfsX5 | Frameshift (2 bp deletion) | Heterozygous (female) | Sanger sequencing | Pathogenic (PVS1 + PM1 + PM2 + PP4) | No | Mother: coarse facial features, hypertelorism, telecanthus, soft hands with short tapered fingers, transverse hypothenar crease, distal axial triradius | Novel mutation; not previously reported |
| Loupe et al., 2014 | USA | Not reported(see below for details *1)) | c.865_866delCA | p.Gln289ValfsX5 | Frameshift (2 bp deletion) | Heterozygous (female) | Sanger sequencing | Pathogenic (PVS1 + PM1 + PM2 + PP4 | No | Proposita (FII-6, 4.5 mo): features of CLS + WS4, hypertelorism, telecanthus, tapered fingers, light blue irises, HSCR, developmental delay | Positive for both CLS and WS4 |
| Loupe et al., 2014 | USA | Not reported(see below for details *1)) | c.865_866delCA | p.Gln289ValfsX5 | Frameshift (2 bp deletion) | Heterozygous (female) | Sanger sequencing | Pathogenic (PVS1 + PM1 + PM2 + PP4 | No | FII-2 (9y): CLS phenotype with fusiform fingers, distal axial triradius, hypertelorism, telecanthus, developmental delay, speech therapy | — |
| Loupe et al., 2014 | USA | Not reported(see below for details *1) | c.865_866delCA | p.Gln289ValfsX5 | Frameshift (2 bp deletion) | Heterozygous (female) | Sanger sequencing | Pathogenic (PVS1 + PM1 + PM2 + PP4 | No | FII-3 (7y): CLS phenotype with proximal axial triradius (L), distal (R), extra finger crease, hypothenar crease, hypertelorism, telecanthus, developmental delay | — |
| Loupe et al., 2014 | USA | Not reported(see below for details *1)) | c.865_866delCA | p.Gln289ValfsX5 | Frameshift (2 bp deletion) | Heterozygous (female) | Sanger sequencing | Pathogenic (PVS1 + PM1 + PM2 + PP4 | No | FII-4 (4y): suspected CLS + WS4, blue eyes, palm crease disruptions, hypertelorism, telecanthus, developmental delay, speech therapy | — |
| Arslan et al., 2014 | Turkey | Exon 21 | c.1973_1974insA | p.M659Dfs*55 | Frameshift | Heterozygous (F) | Sanger sequencing (ABI 3130) | Pathogenic (PVS1 + PM1 + PM2 + PP4 | No | Stimulus-induced myoclonus; classic CLS phenotype | Frameshift, truncating mutation; affects kinase domain; predicted disease-causing; in silico tools used (Mutation taster, Polyphen2, SIFT, MutationAssessor). |
| Matsumoto et al., 2013 | Japan | Multi-exon / whole-gene | — (CNV; not applicable) | — | Microduplication | Hemizygous (male) | Array-CGH | Likely pathogenic | Yes — chrX:19,915,395–20,498,961 (GRCh37) | Male (IV-6, 15y): mild ID (IQ 63), ADHD, panic attacks, localization-related epilepsy (complex partial seizure), normal facial/bony features | RPS6KA3 protein ~2.12× control |
| Matsumoto et al., 2013 | Japan | Multi-exon / whole-gene | — (CNV; not applicable) | — | Microduplication | Hemizygous (male) | Array-CGH | Likely pathogenic | Yes — chrX:19,915,395–20,498,961 (GRCh37) | Male (IV-7, 13y): mild ID (IQ 52), localization-related epilepsy (complex partial seizure), no dysmorphic features | RPS6KA3 protein ~1.91× control |
| Matsumoto et al., 2013 | Japan | Multi-exon / whole-gene | — (CNV; not applicable) | — | Microduplication | Hemizygous (male) | Array-CGH | Likely pathogenic | Yes — chrX:19,915,395–20,498,961 (GRCh37) | Male (IV-10, 4y, different father): borderline developmental level, ADHD | RPS6KA3 protein ~1.53× control |
| Matsumoto et al., 2013 | Japan | Multi-exon / whole-gene | — (CNV; not applicable) | — | Microduplication | Heterozygous (female) | Array-CGH | Likely pathogenic | Yes — chrX:19,915,395–20,498,961 (GRCh37) | Female (IV-8, 11y): unaffected carrier, random XCI pattern (67:33) | RPS6KA3 protein ~2.14× control |
| Matsumoto et al., 2013 | Japan | Multi-exon / whole-gene | — (CNV; not applicable) | — | Microduplication | Heterozygous (female) | Array-CGH | Likely pathogenic | Yes — chrX:19,915,395–20,498,961 (GRCh37) | Female (IV-9, 6y, different father): pervasive developmental disorder (PDD) | HUMARA non-informative; protein level not measured |
| Matsumoto et al., 2013 | Japan | Multi-exon / whole-gene | — (CNV; not applicable) | — | Microduplication | Heterozygous (female) | Array-CGH | Likely pathogenic | Yes — chrX:19,915,395–20,498,961 (GRCh37) | Mother (III-4): depression, normal cognition | RPS6KA3 protein ~1.77× control |
| Tejada et al., 2011 | Spain | Multi-exon / whole-gene | — (CNV; not applicable) | — | Microduplication | Hemizygous (male) | MLPA + array-CGH + FISH + qPCR + immunoblot | Likely Pathogenic (1A, 2A, 4O, 5A) | Yes — arr[GRCh37] Xp22.12(19410850_20460348)x2 | Male (IV-1, 8y): mild ID (IQ 81), psychomotor retardation, mild motor hyperactivity, secondary attention deficit, poor language skills, normal growth, follicular keratosis, mild hypoacusis (resolved post-surgery) | Increased RPS6KA3 mRNA (~2×) and protein |
| Tejada et al., 2011 | Spain | Multi-exon / whole-gene | — (CNV; not applicable) | — | Microduplication | Hemizygous (male) | MLPA + array-CGH | Likely Pathogenic (1A, 2A, 4O, 5A) | Yes — arr[GRCh37] Xp22.12(19410850_20460348)x2 | Male (III-1, 36y): learning difficulties, delayed language in childhood, did not complete primary education | Healthy adult functioning |
| Tejada et al., 2011 | Spain | Multi-exon / whole-gene | — (CNV; not applicable) | — | Microduplication | Hemizygous (male) | MLPA + array-CGH | Likely Pathogenic (1A, 2A, 4O, 5A) | Yes — arr[GRCh37] Xp22.12(19410850_20460348)x2 | Male (III-4, 22y): learning difficulties, delayed language in childhood, did not complete primary education | Healthy adult functioning |
| Tejada et al., 2011 | Spain | Multi-exon / whole-gene | — (CNV; not applicable) | — | Microduplication | Hemizygous (male) | MLPA + array-CGH + qPCR + immunoblot | Likely Pathogenic (1A, 2A, 4O, 5A) | Yes — arr[GRCh37] Xp22.12(19410850_20460348)x2 | Male (IV-2, 5y): learning difficulties noted after school start, less severe than proband | Increased RPS6KA3 mRNA (~2×) and protein |
| Tejada et al., 2011 | Spain | Multi-exon / whole-gene | — (CNV; not applicable) | — | Microduplication | Heterozygous (female) | MLPA + array-CGH + FISH + XCI | Likely Pathogenic (1A, 2A, 4O, 5A) | Yes — arr[GRCh37] Xp22.12(19410850_20460348)x2 | Female (III-2, mother): healthy, normal cognition, random XCI | — |
| Tejada et al., 2011 | Spain | Multi-exon / whole-gene | — (CNV; not applicable) | — | Microduplication | Heterozygous (female) | MLPA + array-CGH + XCI | Likely Pathogenic (1A, 2A, 4O, 5A) | Yes — arr[GRCh37] Xp22.12(19410850_20460348)x2 | Female (II-4, grandmother): healthy, normal cognition, random XCI (49:51) | — |
| Senel et al., 2011 | Turkey | Exon 7 | Not specified | p.Ala180Asp | Missense | Hemizygous (M) | Sanger sequencing | Likely Pathogenic (PM1 + PM2 + PP3 + PP4) | No | Classic CLS phenotype | Novel missense mutation at codon 180. Mother asymptomatic; maternal testing not performed. Array CGH and karyotype normal. |
| Martinez et al., 2011 | USA | Intron 12 | c.1000-2A>G |  | Splice-site | Hemizygous (M) | Sanger sequencing | Pathogenic (PVS1 + PS2 + PM2 + PP4) | No | CLS + LV noncompaction cardiomyopathy with restrictive pattern | Novel splice-site mutation at nucleotide 1000-2, predicted to cause abnormal splicing (11 bp deletion). Maternal testing: de novo mutation. |
| Jurkiewicz et al., 2010 | Poland | Exons 1–16 | c.1-?_1443+?del | p.? | Large deletion | Heterozygous (F) | Sanger + MLPA | Pathogenic (PVS1 + PM2 + PP4) | Yes | Classic CLS phenotype | Large deletion—entire N-terminal + part of C-terminal kinase domains; mother noncarrier; X-inactivation random (52:48). |
| Jurkiewicz et al., 2010 | Poland | Exon 4 | c.262_263insA | p.Ile88AsnfsX5 | Frameshift | Heterozygous (F) | Sanger sequencing | Pathogenic (PVS1 + PM2 + PP4) | No | Classic CLS phenotype | Frameshift—N-terminal kinase domain; mother noncarrier; X-inactivation random (75:25). |
| Jurkiewicz et al., 2010 | Poland | Exon 11 | c.896delT | p.Leu299TyrfsX13 | Frameshift | Heterozygous (F) | Sanger sequencing | Pathogenic (PVS1 + PM2 + PP4) | No | Classic CLS phenotype | Frameshift—N-terminal kinase domain; mother noncarrier; X-inactivation random (68:32). |
| Jurkiewicz et al., 2010 | Poland | Exon 18 | c.1610A>G | p.His537Arg | Missense | Heterozygous (F) | Sanger sequencing | Likely Pathogenic (PM1 + PM2 + PP3 + PP4) | No | Classic CLS phenotype | Missense—C-terminal kinase domain; mother noncarrier; X-inactivation random (72:28). |
